# Healthcare Application of In-Shoe Motion Sensor for Older Adults: Frailty Assessment Using Foot Motion during Gait

**DOI:** 10.3390/s23125446

**Published:** 2023-06-08

**Authors:** Chenhui Huang, Fumiyuki Nihey, Kazuki Ihara, Kenichiro Fukushi, Hiroshi Kajitani, Yoshitaka Nozaki, Kentaro Nakahara

**Affiliations:** Biometrics Research Labs, NEC Corporation, Hinode 1131, Abiko 270-1198, Chiba, Japan; nihey@nec.com (F.N.); kazuki.ihara@nec.com (K.I.); k-fukushi@nec.com (K.F.); h-kajitani@nec.com (H.K.); yoshitaka-nozaki@nec.com (Y.N.); k-nakahara@nec.com (K.N.)

**Keywords:** frailty assessment, in-shoe motion sensor, gait analysis, healthcare application, older adults

## Abstract

Frailty poses a threat to the daily lives of healthy older adults, highlighting the urgent need for technologies that can monitor and prevent its progression. Our objective is to demonstrate a method for providing long-term daily frailty monitoring using an in-shoe motion sensor (IMS). We undertook two steps to achieve this goal. Firstly, we used our previously established SPM-LOSO-LASSO (SPM: statistical parametric mapping; LOSO: leave-one-subject-out; LASSO: least absolute shrinkage and selection operator) algorithm to construct a lightweight and interpretable hand grip strength (HGS) estimation model for an IMS. This algorithm automatically identified novel and significant gait predictors from foot motion data and selected optimal features to construct the model. We also tested the robustness and effectiveness of the model by recruiting other groups of subjects. Secondly, we designed an analog frailty risk score that combined the performance of the HGS and gait speed with the aid of the distribution of HGS and gait speed of the older Asian population. We then compared the effectiveness of our designed score with the clinical expert-rated score. We discovered new gait predictors for HGS estimation via IMSs and successfully constructed a model with an “excellent” intraclass correlation coefficient and high precision. Moreover, we tested the model on separately recruited subjects, which confirmed the robustness of our model for other older individuals. The designed frailty risk score also had a large effect size correlation with clinical expert-rated scores. In conclusion, IMS technology shows promise for long-term daily frailty monitoring, which can help prevent or manage frailty for older adults.

## 1. Introduction

### 1.1. Background

**Figure 1:**
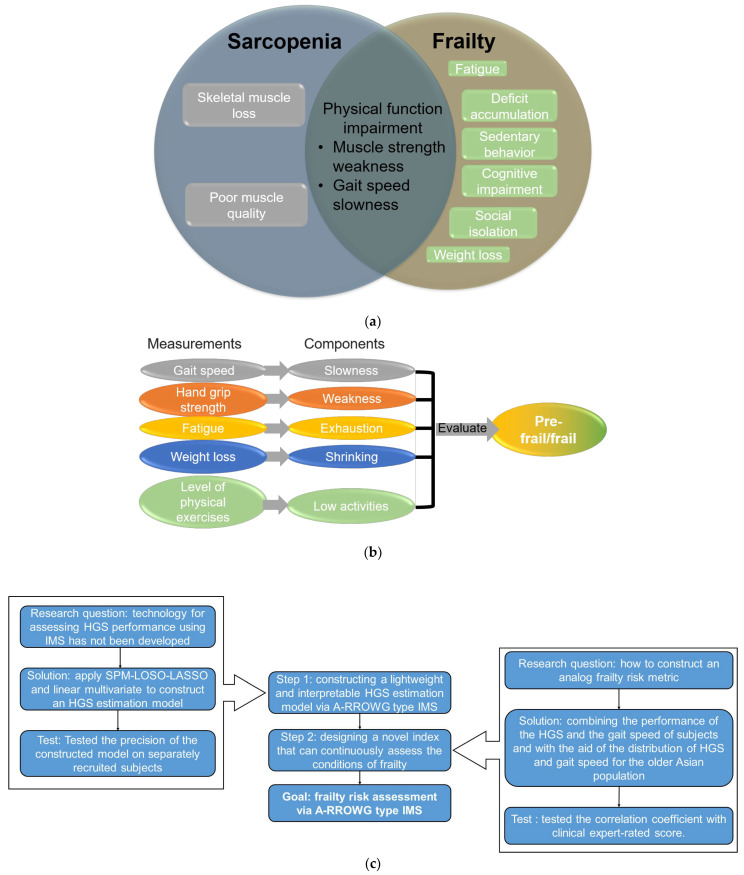
(**a**) Relationship between sarcopenia and frailty. (**b**) Revised Japanese version of Cardiovascular Health Study criteria. (**c**) Diagram which explains the development process of achieving frailty risk assessment via A-RROWG-type IMS.

Typically, skeletal muscle mass begins to decline gradually at around age 45, after reaching its peak in the early adult years [1]. Additionally, gait speed, which has been deemed the sixth vital sign [2], significantly decreases in older adults after age 60 [3]. The decline in skeletal muscle mass and gait speed below a critical threshold may result in physical functional impairments that limit mobility, such as walking, climbing stairs, and crossing over obstacles [4]. These impairments may lead to sarcopenia or frailty in older adults [5] (see Figure 1a).

Although the relationship between sarcopenia and frailty has yet to be fully characterized, these conditions share many commonalities. Both are linked to physical functional impairment, and sarcopenia is an age-related, long-term process that involves the loss of muscle mass and strength, affecting mobility and nutritional status [6,7,8]. Additionally, physical frailty may result in sedentary behavior, cognitive impairment, and social isolation [9]. Frailty is closely associated with various detrimental outcomes for older adults, such as an increased risk of falls and fractures, impaired ability to perform daily activities, loss of independence, the need for long-term care placement, and even death [10,11,12]. Fortunately, appropriate exercise and nutritional treatment can postpone, recover, and effectively manage frailty, especially if the frail condition can be assessed in daily living [13].

The Asian Working Group on Sarcopenia (AWGS) defines sarcopenia for Asian individuals and includes criteria for assessing muscle function in diagnosing sarcopenia [14]. These criteria consist of gait speed measurement and hand grip strength (HGS) measurement, which are the simplest well-validated protocols for assessing muscle function in clinical practice [15]. The threshold for HGS measurement is 28 kg for males and 18 kg for females, while the gait speed requirement for both sexes is 1.0 m/s. These criteria are also included in the revised Japanese version of the Cardiovascular Health Study (J-CHS) criteria for diagnosing physical pre-frailty/frailty [16] (see Figure 1b). In addition, three other criteria are evaluated subjectively by the participants themselves using a questionnaire. Participants rate their conditions on a scale from 0 to 2, and those with scores higher than 2 are categorized as “Robust”, “Pre-frail”, or “Frail”.

The assessments mentioned above generally require older adults to visit specialized facilities and undergo evaluations under the supervision of clinicians. However, in certain areas, particularly rural regions in Japan where healthcare resources are limited, monitoring older adults’ body conditions can be challenging. Moreover, for urban senior citizens, weekly or monthly facility visits are not always feasible as they can increase the burden on seniors and healthcare systems alike. The recent development of Internet of Things (IoT) technologies for healthcare [17,18,19] has introduced wearable technologies as a viable option to monitor pre-frailty/frailty in daily living. By monitoring physical performance in daily living over a long period, wearable technologies can help alert users to seek further examination or appropriate treatments on demand, prevent or manage the progression of frailty, and ultimately reduce the burden on healthcare systems. 

A new approach to assessing pre-frailty/frailty has been introduced, proposing that wearable sensors can enable the simple monitoring of gait parameters (GPs), including gait speed, during daily walking. This has made gait speed monitoring “smart”, as all data processing can be conducted on an edge device [20,21,22]. However, HGS assessment remains challenging for many individuals, as it requires clinicians to perform assessments in a facility setting, following specific protocols [15]. Despite this, IoT technologies for HGS assessment have been developed by researchers such as Becerra et al. [23], who developed a wireless hand grip device for force analysis, Chen et al. [24], who proposed a hand rehabilitation system with an HGS assessment function via soft gloves, and Wang et al. [25], who developed a novel flexible sensor to assess HGS. Nevertheless, for many users, wearing sensors on their hands may be inconvenient and challenging, considering daily routines. This may pose an obstacle to achieving reliable monitoring of frailty in daily living.

Smart shoes/insoles with motion sensors have been proposed to improve the practicality of daily gait analysis. These devices are considered promising in various healthcare applications that require daily gait analysis, including Parkinson’s disease, gait rehabilitation, and foot deformity detection [26,27,28]. In this paper, we refer to this type of smart motion sensor as an “in-shoe motion sensor” (IMS). An IMS can easily and noninvasively gather abundant information related to gait kinematics, including gait speed, stride length, stance phase duration, instantaneous linear and rotational foot motion, and 3-D foot angular posture [26,29,30]. Furthermore, IMSs can be placed in various types of shoes or insoles, making them an unobtrusive addition to daily life.

We considered developing a frailty risk assessment method using only an IMS as a user-friendly solution for daily frailty assessment with the following benefits: (1) helping users avoid the burden of wearing multiple sensors and (2) simplifying the wearable sensor system for frailty assessment. To achieve this goal, we identified two necessary steps: (1) constructing an HGS estimation model using foot motion data obtained from an IMS and (2) designing a novel index capable of continuously assessing the conditions of frailty. In the subsequent sections, we provide a detailed explanation of these two steps.

### 1.2. Step 1 to Goal: Constructing HGS Assessment Model on an IMS and Related Work

#### 1.2.1. Research Question in Step 1

Generally, IMSs can transmit detailed waveforms wirelessly to a smartphone or server for further analysis, which consumes a significant amount of power. As a result, these IMSs need to be frequently charged, reducing their usability for practical applications. In a previous study, we developed a new type of IMS, which is small and lightweight, can be attached to insoles, and has optimally designed power-saving operation sequences and modes for practical applications. Our study showed that this IMS achieved high usability for long-term daily measurement without the need for battery charging for up to one year [29]. One key feature contributing to power savings is that our IMS can perform simple data processing and calculate common spatiotemporal GPs, such as gait speed, stride length, and stance phase duration, using inertial measurement unit (IMU) signals. We have named this type of IMS A-RROWG^Ⓡ^. These features enable A-RROWG to collect daily gait data over long periods, regardless of location and time, without the user noticing the sensor’s presence. The research question for Step 1 is how to construct an HGS assessment model that is feasible for an A-RROWG-type IMS and that can be proven effective. However, to the best of our knowledge, no technology has been developed for assessing HGS performance using IMSs.

#### 1.2.2. Ideas for Solving the Research Question in Step 1

Due to the characteristics of A-RROWG, the HGS assessment model must be lightweight enough to be implemented on it. Therefore, rather than applying recent machine learning methods that require a large computation capacity [31], we focused on developing a lightweight, high-precision estimation model via linear multivariate regression with a minimum number of predictors required. This development included two tasks: (1) identifying predictors that highly correlate with HGS and (2) reducing redundant predictors via feature selection. 

Gait speed has been suggested to correlate with HGS [32,33], indicating that gait features might be a useful predictor for HGS assessment. However, gait speed is not a specific predictor for HGS as it can also be influenced by other factors, such as knee osteoarthritis [34] or depression [35], making it challenging to construct an accurate model.

To address this limitation, we proposed considering additional potential predictors for HGS assessment. Previous research has demonstrated that HGS correlates with knee extension muscles, specifically the quadriceps [36,37], which play a crucial role in walking. Since gait is a periodic movement, the same motions using muscles are repeated during specific gait phases in every gait cycle (GC). Although the quadriceps do not directly control foot motion, they should impact foot motion through their control of the knee joint and lower leg. Therefore, we considered predictors for HGS assessment that can be determined from foot motion signals during specific gait phases, specifically those gait phases where the quadriceps are activated. 

For the second task of selecting appropriate predictors, several techniques, such as LASSO [38], Bayesian methods such as Bayesian LASSO [39], deep learning methods for sparse learning [40], and multi-objective optimization methods [41], have been proposed. However, multi-objective optimization methods are suitable for optimizing multiple conflicting objectives simultaneously, which is not within the scope of linear regression methods utilized in our study. LASSO and Bayesian LASSO are more feasible alternatives, but Bayesian LASSO may require more substantial expertise to interpret results accurately. As such, we chose to apply LASSO for feature selection. 

In conventional LASSO, cross-validation approaches [42] are commonly used to select the LASSO tuning parameter value. However, these techniques typically consider randomly selecting training and validation sets without considering variations between individuals. To ensure model robustness and account for individual differences, we combined LASSO with a leave-one-subject-out (LOSO) process. This approach involved running multiple LASSO analyses by looping the LOSO process for all subjects, conceptually similar to jackknife, resampling method [43], to approximate the nature of the population estimator and improve model robustness against individual differences. 

In our previous studies, we developed an algorithm capable of automatically extracting novel significant gait predictors from foot motion, selecting optimal features, and constructing an assessment model, valid for estimating adults’ foot function and older adults’ balance ability measured by the outcome of a functional reach test (FRT) [44,45]. In this study, we constructed an HGS estimation model using this algorithm via the following steps:

1. Identifying significant gait phases with statistically significant correlation with the target variable using statistic parametric mapping (SPM) [46], which was proven effective in biomechanical studies. The significant gait phases always continuously appeared, performing as clusters on the temporal axis; thus we called them, “gait phase clusters” (GPCs).
2. Conducting predictors by averaging the foot motion signals in the GPCs to obtain IMS predictors that can be implemented on the A-RROWG-type IMS. Although there are clustering algorithms, such as community detection algorithms [47], due to the temporal continuity of foot motion, using the integral average of the signals in GPC as a single predictor is sufficient and helpful for implementation on the A-RROWG-type IMS.
3. Reducing redundant predictors and selecting appropriate predictors using our original algorithm, the leave-one-subject-out least absolute shrinkage and selection operator (LOSO-LASSO).
4. Constructing a multivariate linear regression estimation model.

We refer to our approach as SPM-LOSO-LASSO, which aids in constructing a biomechanically interpretable HGS estimation model that is both lightweight enough for implementation on an edge device and precise in its predictions. In a previous study, we demonstrated the construction and operation of the IMS predictors on an A-RROWG-type IMS [44]. In this study, we have incorporated individual physical attributes (IPAs), such as age, height, weight, and body mass index (BMI), and designed GPs, including previously proposed temporal and spatial GPs (we list them in Section 2.4), as auxiliary predictors to enhance the model’s precision. Considering the gait variance between biological sexes [48], we have constructed separate estimation models for males and females. Some of our findings in this report are based on the work presented at the 44th International Conference of the IEEE Engineering in Medicine and Biology Society (EMBC 2022) [49]. 

### 1.3. Step 2 to Goal: Related Work on Frailty Assessment and Designing a Frailty Risk Score

#### 1.3.1. Research Question in Step 2

Aside from the cardiovascular health study (CHS) criteria, there are alternative methods for diagnosing frailty in clinical practice. Examples include the phenotype model [50] and accumulated deficit model [51]. To assess frailty levels in daily living, several techniques based on wearable sensor measurements have been proposed [22]. For instance, using wearable motion sensors, Schwenk et al. [52] conducted home assessments of established gait outcomes to identify pre-frailty and frailty. Razjouyan et al. [53] utilized a pendant motion sensor to develop a composite model for discriminating three frailty categories: non-frail, pre-frail, and frail. In addition, Greene et al. [54] aimed to create an automatic, non-expert quantitative assessment of the frailty state based on wearable inertial sensors.

However, previous research studies focused solely on discriminating two or three frailty levels. The transition from non-frail to pre-frail or pre-frail to frail is a gradual, long-term process. According to a previous study [55], the pooled incidence rate of pre-frailty was 15.1%, and that of frailty was 4.3% based on multiple cohort studies. Given that body performance tends to decline with age in the absence of intervention, it is reasonable to hypothesize that the higher the current condition’s frailty risk, the greater the likelihood of future deterioration. To assist users in delaying and managing frailty progression adequately, merely classifying frailty levels is considered insufficient. Consequently, the research question in Step 2 is how to construct an analog frailty risk metric and demonstrate its effectiveness. 

#### 1.3.2. Ideas for Solving the Research Question in Step 2

An analog frailty risk score could prove beneficial for various reasons, such as providing users with an intuitive representation of their body condition’s long-term changes, enabling a more comprehensive user rating, and demonstrating the effects of exercise. Given that HGS and gait speed are critical factors in current frailty assessment, we assert that their performance must feature significantly in frailty risk assessment. Consequently, we developed a frailty risk score in this study by merely combining the HGS and gait speed performance of the subjects. Moreover, we utilized the HGS distribution [56] and gait speed data for the Asian population aged over 60 years [57,58] to design our frailty risk score.

### 1.4. Testing Constructed HGS Estimation Model and Frailty Risk Score

After constructing and validating the model, we conducted two separate tests on a group of older healthy adults who were recruited independently from those used for constructing the model. 

The first test involved examining the precision of the HGS assessment model on the separately recruited subjects. 

The second test involved testing the effectiveness of our original frailty risk score, which was used to demonstrate the possibility of evaluating frailty via IMSs in subjects who were also recruited separately. These subjects were rated using a continuous score ranging from 0 to 100 by experts, including clinicians and physiotherapists with over 5 years of experience, who observed their gait. The score served as a reference for their risk of frailty. We tested the correlation coefficient between the designed score and the expert-rated score.

### 1.5. The Development Process and Main Contributions in this Study

In summary, Figure 1c presents a diagram that outlines the development process of achieving frailty risk assessment via the A-RROWG-type IMS. The main contributions of this study are as follows:

(1) We discovered novel predictors for HGS assessment obtained from foot motions.
(2) We constructed a lightweight HGS assessment model that can be feasibly implemented in the A-RROWG-type IMS, which serves as a key module for long-term frailty assessment.
(3) We tested the effectiveness and robustness of the constructed model using a group of separately recruited subjects.
(4) We designed an analog frailty risk score and evaluated its effectiveness for frailty risk assessment via an IMS.

The acronyms and symbols used in this manuscript can be referenced in Table A1 in Appendix A. 

## 2. Materials and Methods

### 2.1. Subjects and Their Characteristics

To contribute to potential applications for frailty prevention, as well as postponing and managing its progression, we recruited healthy older adults who could participate in the experiment independently. We recruited three separate groups of healthy older subjects with different ages, heights, and weights for model construction (Group I), Test 1 (Group II+III, combining data in Group II and Group III together), and Test 2 (Group III). We successfully collected data from 62 subjects (27 males and 35 females) for Group I, 20 females for Group II, and 25 subjects (6 males and 19 females) for Group III. All subjects were able to walk independently without assistive devices, had no history of severe neuromuscular or orthopedic diseases, had normal or corrected-to-normal vision, and had no communication obstacles. After explaining the experimental procedure to the subjects, we obtained their informed consent before the experiment. This study received approval from the NEC Ethical Review Committee for Life Sciences (Approval No. LS2021-004, 2022-002) and the Ethical Review Board of Tokyo Medical and Dental University (Approval No. M2020-365). The demographic data are summarized in Table 1, with HGS and gait speed serving as reference values.

The average age of both male and female subjects in all three groups was over 70 years old. Although the average BMIs indicate that most subjects had a normal body mass, we ensured that subjects with a wide range of body mass were recruited, including those with maximum and minimum BMIs. In Group I, male and female subjects had similar age characteristics (*p* = 0.755), and no significant sex difference in gait speed was found (*p* = 0.453). The data also show that the female subjects for model construction (Group I) were similar in age to those for model testing (Group II+III) (*p* = 0.604), as well as in terms of HGS and gait speed (HGS: *p* = 0.395; gait speed: *p* = 0.265). However, compared with the male subjects in the two groups, the age in Group II+III was higher than that in Group I (*p* = 0.040). Although there was no significant difference in gait speed (*p* = 0.052), due to age, the HGSs in Group II+III were much lower (*p* = 0.021). When comparing female subjects in Groups I and III, no significant differences in age, HGS, and gait speed were found between them (*p* = 0.058, 0.102, 0.972). According to the J-CHS scores of the subjects in Group III, 60% of the subjects self-assessed themselves as not being frail, and none of them assessed themselves as frail. Further details on how the J-CHS scores were calculated for the subjects are presented in Section 2.2.

### 2.2. Experiment

To achieve our final goal, we collected five types of data from subjects in an indoor environment, performing the following steps:

Step 1 At the start of the experiment, all subjects were asked to complete a questionnaire to provide basic information, including age, height, and weight, and for the calculation of BMI based on their answers.
Step 2 The same questionnaire included four questions based on the J-CHS criteria:
  Q1. Have you lost more than 2–3 kg in the past 6 months?
  Q2. In the past two weeks, have you felt tired for no reason?
  Q3. Do you engage in light exercise or gymnastics at least once a week?
  Q4. Do you engage in regular exercise or sports at least once a week?

From the four questions, we calculated the J-CHS score for each subject as subjective frailty reference data.

Step 3 After answering the questionnaire, subjects were guided to measure their HGS, which served as the reference HGS value in this study.
Step 4 Subjects were asked to walk in a straight line. In this step, we collected foot motion data for calculating GPs and IMS predictors, as well as reference gait speed data for all subjects. Additionally, for those in Group III, video recordings were made while they walked.
Step 5 We sent the walking videos to clinical experts to obtain expert-rated frailty risk scores as objective frailty reference data.

Further details on Steps 2 through 5 are explained in the following subsections.

#### 2.2.1. Step 2 of the Experiment

For Q1 and Q2, one point was added for each “yes” answer. For Q3 and Q4, if both questions were answered with “no”, one point was added, and if either was answered with “yes”, no points were added. The total J-CHS score was obtained by totaling the points. Finally, we checked whether the reference HGS and gait speed were below the threshold specified in the J-CHS criteria to determine the subjects’ total J-CHS score. Subjects who scored 0, 1–2, and higher than 2 were classified as “Robust”, “Pre-frail”, and “Frail”, respectively.

#### 2.2.2. Step 3 of the Experiment

**Figure 2:**
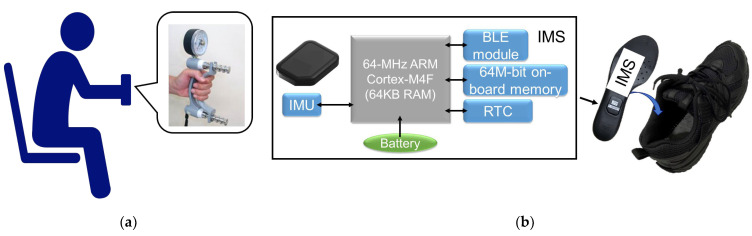
Schematic of (**a**) measurement of HGS. The subjects were asked to sit on an armchair sitting with the elbow in 90° flexion, but the elbow cannot touch the chair arms. The dynamometer was set at handle position “two”. (**b**) The structure of an IMS (left side). IMS was embedded in an insole placed under the foot arch near the calcaneus side and then inserted into a sport shoe.

To assess the HGS of the subjects, we used a Jamar hydraulic hand dynamometer (Lafayette Instrument Company, Lafayette, IN, USA). The measurement process followed the method suggested in a previous study [15], as shown in Figure 2a. Subjects were asked to sit on an armchair with their elbow flexed at 90°, without touching the chair arms. The Jamar is a variable hand-span dynamometer with five handle positions. The dynamometer was set to handle position “two”, and both hands were measured three times with subjects exerting their best effort. To determine the representative HGS of each subject, we calculated the mean value of the six measurements. This mean value served as the reference value for HGS in this study.

#### 2.2.3. Step 4 of the Experiment

To collect foot motion data, the subjects were asked to walk straight along 16 m lines for four trials at a self-determined comfortable speed. Before data collection, they were given a 2-min practice session to familiarize themselves with the environment and procedure. While walking, their foot motions were recorded by two IMSs embedded in insoles placed under the arches of both feet near the calcaneus side (see Figure 2b). This placement ensured that the subjects could walk comfortably. Please note that during the feasibility study stage of this study, foot motion data were temporarily recorded onto the onboard memory during experiments and would later be transferred to a personal computer for data processing. The characteristics of the IMS are described in Section 2.3. 

The time taken by each subject to walk 10 m along the 16 m lines was recorded using a digital stopwatch to calculate their average gait speed when walking at a uniform pace. This speed was treated as the reference value for gait speed in this study. Subjects in Group III were also recorded while walking by two video cameras placed at the side and end of the walking path. To protect their privacy, their faces were obscured.

#### 2.2.4. Step 5 of the Experiment

After gait data collection was finished, the videos were sent to six clinical experts in gait evaluation. They were asked to score the subjects regarding the risk of “being diagnosed with frailty within the next 5 years” on a 100-point scale by observing their gait. A subject considered by the rater to have the highest risk was rated as 100, and a subject with the lowest risk was rated as 0. The relative frailty risk of the remaining subjects compared with the highest and lowest ones was scored between 0 and 100. Then, every subject had six scores. Except for observing the recorded videos, the raters were not given any personal information about the subjects.

### 2.3. Characteristics of IMS

The IMSs used in this study have the same structure as A-RROWG-type IMSs. Each IMS consists of a 6-axis IMU (BMI 160, Bosch Sensortec, Reutlingen, Germany), an ARM Cortex-M4F microcontroller unit (MCU) with Bluetooth module (nRF52832, CPU: 64 MHz, RAM: 64 KB, ROM: 512 KB, Nordic Semiconductor, Oslo, Norway), onboard memory (AT45DB641, 64 Mbit, Adesto Technologies, Santa Clara, CA, USA), a real-time clock (RTC) (RX8130CE, EPSON, Suwa, Japan), a control circuit, and a 3V coin lithium-ion battery (CLB2032 T1, 300 mAh, Maxell, Tokyo, Japan). The device is lightweight (12 g, including the coin battery) and compact (29 mm × 40 mm × 7 mm) enough to be placed at the arch of the foot. Please note that during the feasibility study stage, the IMSs were set to developer mode, which differed from A-RROWG in that all calculations were performed on the device. Under this mode, raw foot motion waveform data were first recorded on the IMSs’ onboard memory and then sent to a PC via Bluetooth after the experiment. We developed dedicated software for controlling data recording start and end in the IMSs and for downloading raw data from the onboard memory of the IMSs to a PC via Microsoft Visual Studio (Microsoft, Redmond, WA, USA). 

The IMSs can directly measure three axes of acceleration, *A_x_* (medial: +, lateral: −), *A_y_* (posterior: +, anterior: −), and *A_z_* (superior: +, inferior: −), as well as those of angular velocity, *G_x_* (sagittal plane (*Y–Z*): plantarflexion: +, dorsiflexion: −), *G_y_* (frontal plane (*X–Z*), eversion: +, inversion: −), and *G_z_* (horizontal plane (*X–Y*), internal rotation: +, external rotation: −). Inside the IMSs, the three axes of sole-to-ground angles (SGAs), *E_x_* (roll angle, plantarflexion: +, dorsiflexion: −), *E_y_* (pitch angle, eversion: +, inversion: −), and *E_z_* (yaw angle, internal rotation: +, external rotation: −), were calculated using a Madgwick filter [59]. Specifically, the acceleration values were corrected to the global coordinates in each independent trial. The IMSs had a data sampling frequency of 100 Hz, and their measurement range for acceleration was ±16 g, while that for angular velocity was ±2000 degrees/s.

### 2.4. Signal Processing and GPs

For all data processing, simulation, and model construction tasks, MATLAB (MathWorks, Natick, MA, USA) was used in this study. 

**Figure 3:**
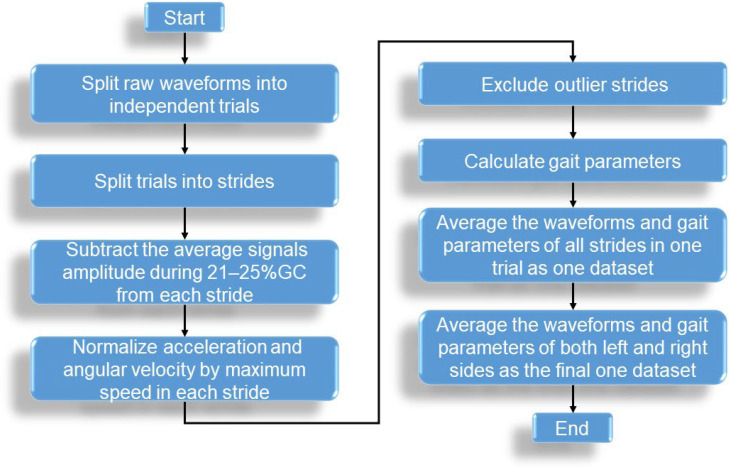
The flowchart of procedures to obtain GP predictors.

To construct the HGS estimation model via the SPM-LOSO-LASSO algorithm, predictors from three categories were required: IPAs, temporospatial GPs, and IMS predictors. Temporospatial GPs and IMS predictors were obtained by processing one stride of the foot motion waveform. In this section, we explain the procedures used to obtain GP predictors. The flow chart is shown in Figure 3. 

During the preliminary stage, two primary tasks were completed. The first task involved processing every stride of the foot motion waveform into data matrices. The second task focused on calculating the GPs that were extracted from each stride of the foot motion waveform. The third task was to obtain a set of average foot motion waveforms and GPs in each trial. 

For the first task, to prepare the nine-dimensional foot motion signals from the IMSs for analysis, the signals were partitioned into individual strides by detecting a heel-strike (HS) event [60]. The IMS signal during the stance phase was then temporally normalized to a 1–60% gait cycle (%GC), while the swing phase was normalized to 61–100%GC to create a 9 × 100 matrix. To eliminate potential biases, we subtracted the average signal amplitude during 21–25%GC from each stride assuming that these phases, where the foot sole fully touches the ground, can be represented as a neutral posture. Additionally, to exclude any walking velocity bias in foot motion, we normalized the amplitude of acceleration and angular velocity waveform of each stride using the corresponding maximum instantaneous velocity during a stride. The instantaneous walking velocity was computed by integrating *A_y_* from a neutral posture to the end of the stride. It is worth noting that we excluded the first and last three strides of each trial, as they were not uniform in speed. Furthermore, we removed any gait outliers from the remaining strides of each participant, following the exclusion criteria outlined in [61]. 

Before temporal normalization, we derived 20 temporal and spatial GPs [29,62] from each stride of the foot motion waveform using the algorithm depicted in [29,62]. These parameters are listed in Table 2. GP01, GP05, and GP06 were normalized by subject height. GP11-14, GP19, and GP20 were normalized by the duration of one stride. GP15, GP16, and GP18 were normalized by the maximum instantaneous walking velocity during the swing phase. 

We then calculated the average foot motion and GPs for each trial on the left and right feet for each subject. The data of the left and right feet were further averaged within each trial. This resulted in each participant having four sets of average foot motions and GPs. Thus, a total of 108 and 140 datasets were generated for males and females in Group I, respectively, and 24 and 156 datasets were generated in Group II+III for males and females, respectively. These processed average waveforms were used to determine new predictors for HGS estimation.

### 2.5. SPM-LOSO-LASSO, Model Evaluation of HGS, and Precision Evaluation of Gait Speed

#### 2.5.1. The Details of SPM-LOSO-LASSO

**Figure 4:**
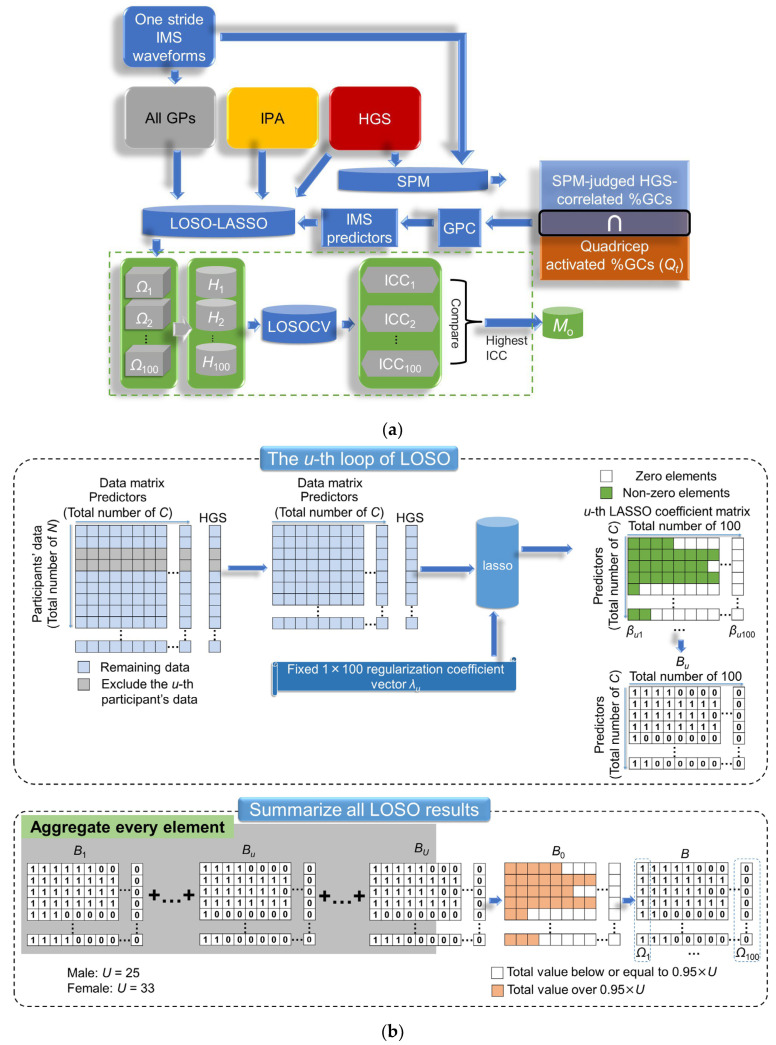

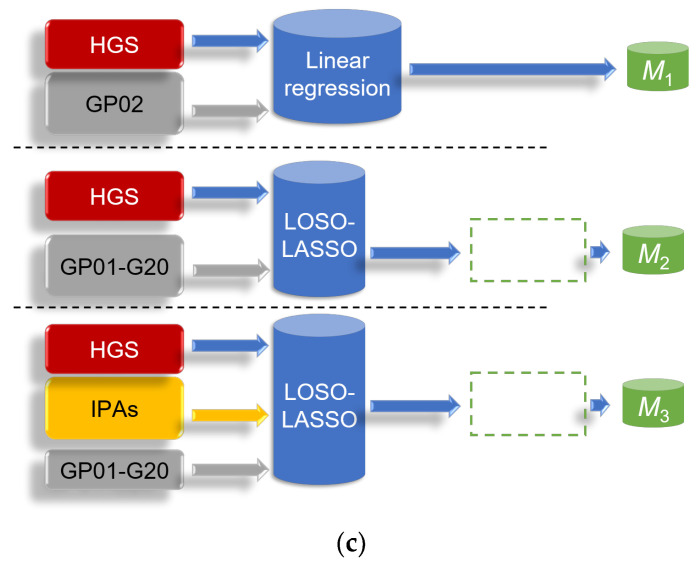
(**a**) Process of feature construction, feature selection, and model construction for HGS estimation. Ω_1_–Ω_100_: 100 types of features combinations in accordance with different regularization coefficients set in LASSO for HGS estimation; *H*_1_–*H*_100_: 100 types of candidate multivariate regression models for HGS estimation; ICC*_k_* denotes ICC value of model *H_k_*; *M_o_*: optimal models for HGS estimation. (**b**) Details of LOSO-LASSO; *U*: total number of participants for training data; *λ_u_*: *u*-th regularization coefficient vector for LASSO, 100 dimensions; *λ_ui_*: *i*-th element of *λ_u_*; *β_ui_*: fitted least-squares regression coefficients corresponding to *λ_ui_*; *B_u_*: *u*-th label matrix obtained by substituting nonzero elements in LASSO coefficient by 1; *B*_0_: label counter matrix; *B*: final label matrix obtained by substituting elements over and below 0.95 × *U* by 1 and 0 in *B*_0_. (**c**) Other three models derived by optimizing three other predictor combinations by the same process as *M_o_*, *M*_1_: gait speed (GP02), *M*_2_: *M*_1_ plus other GPs in one stride, and *M*_3_: *M*_2_ plus IPAs. Green dashed boxes in (**c**) indicate the corresponding process included in the same box shown in (**a**).

In this section, we explain the process of constructing and selecting predictors for HGS estimation via SPM-LOSO-LASSO, following the steps depicted in Figure 4a [44]. Here, IMS predictor processing is part of SPM-LOSO-LASSO. 

To construct IMS predictors from foot motion signals that are significantly correlated with HGS outcome, it is necessary to determine the %GCs that have a significant correlation. For this purpose, we used SPM, a widely used and effective method in biomechanical studies [46,63]. We performed SPM analysis to evaluate the correlation between HGS outcomes and foot motion signals at each %GC. SPM for correlation analysis is a stepwise process. First, a canonical correlation analysis (CCA) with SPM (SPM-CCA) was performed [46]. The %GCs whose test statistic in the CCA exceeded a critical test statistic threshold calculated in accordance with the random field theory (RFT) [64] were determined as significant %GCs. The level of significance was set as *p* < 0.05. Second, as a post hoc test, only data in significant %GCs were further investigated by Pearson’s correlation (PeC) analysis with SPM (SPM-PeC) for each component of the foot motion signal. For each component, the %GCs whose test statistic in the PeC exceeded an RFT-based critical test statistic threshold were judged as the final HGS-correlated significant %GCs for each component. Because there were nine components in the foot motion signals, we conducted Šidák correction [65] at a level of correlation significance where *p_c_* < 0.0057.

Based on biomechanical knowledge, we limited the predictors to the range of approximately 1–16%GC, 48–70%GC, and 92–100%GC, where the quadriceps are mostly activated. These defined quadricep-activation %GCs were used as a filter, denoted as *Q_t_*. The intersection between the %GCs judged by SPM to be HGS-correlated and the *Q_t_* was taken to exclude the %GCs not related to quadricep activities. The intersections were treated as GPCs. The integral average of the signal in GPCs was then used as an IMS predictor, as expressed by (1).

(1) $$C_{i}=\left\{\begin{array}{c} \overset{T_{e}-\Delta T}{\underset{T_{s}}{\sum }}\left(\frac{W\left(T\right)+W\left(T+\Delta T\right)}{2\left(T_{e}-T_{s}\right)}\Delta T\right),\;\;+T_{e}-T_{s}>0\\ \;\;W\left(T_{e}\right),\;+T_{e}-T_{s}=0 \end{array}\right.$$

where *C_i_* means the *i*-th IMS predictor; *T_s_* and *T_e_* mean the start and end of %GCs of GPCs, respectively; and *W* means the waveform of the foot motion signal, where *W* ∊ {*A_x_*, *A_y_*, *A_z_*, *G_x_*, *G_y_*, *G_z_*, *E_x_*, *E_y_*, *E_z_*}.

After collecting the subjects’ IPAs, GPs, and IMS predictors, we formed predictor candidates for model construction. We used our original algorithm, LOSO-LASSO [44], along with the “lasso” function in MATLAB to determine the best selection of predictors. We obtained multiple LASSO analysis results by looping the LOSO process for all subjects. By statistically analyzing these results, we can approximate the nature of the population estimator and thereby make the LASSO analysis more robust against individual differences.

The details of LOSO-LASSO are shown in Figure 4b. In the *u*-th LOSO process, the data of the *u*-th subject are first excluded, and the remaining data are then subjected to LASSO analysis. LASSO solves the following problem:

(2) $$\underset{\beta_{i0},\beta_{i}}{\min}\left(\frac{1}{2N}\overset{N}{\underset{k=1}{\sum }}{\left(y_{k}-\beta_{i0}-x_{k}^{T}\beta_{i}\right)}^{2}+\lambda_{i}\overset{C}{\underset{j=1}{\sum }}\left|\beta_{ij}\right|\right)$$

Here, *N* is the amount of data. *y_k_* is the target variable. *x_k_* is the predictor vector of length *C*. *λ_i_* is a non-negative regularization parameter input to LASSO, which can be set freely. *β_i_* is the set of fitted least-squares regression coefficients, and *β_i_*_0_ is the residual of the linear regression *y_k_* = *x_k_^T^β_i_* + *β_i_*_0_, corresponding to *λ_i_*, which is also the output of LASSO. *β_ij_* is the *j*-th element of *β_i_*. As *λ_i_* increases, the number of nonzero components of *β_i_* decreases. For optimizing feature selection, we set 100 different *λ_i_*’s which formed a geometric sequence to compose a regularization parameter 100-dimensional vector *λ*; thus, the index *i* here means the *i*-th element of *λ*. In each LOSO, 100 *β_i_*’s formed a coefficient matrix. Then, we substituted nonzero elements in LASSO coefficient matrices with 1 to form label matrix *B_u_*.

This process is repeated for each subject. After completion of the LOSO process, we can obtain *U* sets of *B_u_*’s. By summing all *B_u_*’s, we obtain a matrix with a total counter *B*_0_. The elements over 0.95 × *U* (25 for males and 33 for females) in this matrix are substituted with 1, while the remaining elements are substituted with 0, forming the final label matrix *B*. LOSO-LASSO generates 100 types of predictor combinations (denoted as Ω_1_–Ω_100_) based on different regularization coefficient sets in LASSO. Using these features, 100 different candidate multivariate regression models can be obtained for the dataset. We evaluated 100 candidate models (*H*_1_–*H*_100_) for estimating HGS using leave-one-subject-out cross-validation (LOSOCV) and the intraclass correlation coefficient (ICC) of type (2, 1) as the evaluation index, denoted as ICC(2, 1). The model with the highest ICC(2, 1) value was chosen as the optimal model (*M_o_*).

#### 2.5.2. Model Evaluation of HGS and Precision Evaluation of Gait Speed

After selecting *M_o_*, we used LOSOCV to evaluate the degree of agreement and precision between the reference and estimated HGS, using the ICC(2, 1) and mean absolute error (MAE). Additionally, we evaluated the adjusted coefficient of determination (*R*^2^) for the multivariate regression models using all training data (not LOSOCV) and the Pearson’s coefficient of correlation (*r*) between predictors and the outcome of HGS. For comparison, we derived models by optimizing three other patterns of predictor combinations in the same process: *M*_1_ (gait speed (GP02)), *M*_2_ (*M*_1_ plus other GPs in one stride), and *M*_3_ (*M*_2_ plus IPAs) (see Figure 4c).

We evaluated the average value of gait speed measured by the IMS in one trial and used ICC(2, 1) and MAE to assess the agreement and precision between the reference and measured values. 

The guidelines for interpreting ICC inter-rater agreement are as follows: excellent (>0.750), good (0.600–0.749), fair (0.400–0.599), and poor (<0.400) [66]. The guidelines for interpreting *R*^2^ are as follows: none (<0.02), small (0.02 to 0.13), medium (0.14 to 0.26), and large (>0.26). The guidelines for interpreting r are as follows: none (<0.100), small (0.100 to 0.299), medium (0.300 to 0.499), and large (>0.499) [67].

### 2.6. Designing Frailty Risk Score

We assumed that the distribution of HGS and gait speed of our subjects would follow a normal distribution similar to that of the population of older Asian adults. According to [56], the mean values of HGS for males (*N* = 12,190) and females (*N* = 14,154) over 60 years old are 34.7 and 21.9 kg, respectively, and the standard deviations are 7.1 and 4.8 kg, respectively. In [57], the baseline demographic and health characteristics of 1686 community-dwelling Japanese were demonstrated, and no significant difference in gait speed was observed between sexes. Thus, the calculated mean value and standard deviation of gait speed for all subjects were 1.29 and 0.24 m/s, respectively. 

We utilized a probability-distribution-based method to design the frailty risk score. First, we calculated the Z-score of the HGS performance of males and females using Equations (3) and (4), respectively, and that of gait speed using Equation (5), using the mean value and standard deviation of HGS and gait speed for older Asian adults in [56,57].

(3) $$Z_{HGS\_m}=\left(HGS_{m}-34.7\right)/7.1$$

(4) $$Z_{HGS\_f}=\left(HGS_{f}-21.9\right)/4.8$$

(5) $$Z_{GS}=\left(GS-1.29\right)/0.24$$

Here, *HGS_m_*, *HGS_f_*, and *GS* are the HGS of the male subjects, the HGS of the female subjects, and the gait speed of all subjects (no sex difference). *Z_HGS_m_* and *Z_HGS_f_* denote the Z-scores of the HGS performance of males and females, and *Z_GS_* denotes the Z-scores of the gait speed performance for the standard normal distribution. 

Because Z-scores can theoretically be from −∞ to +∞, to constrain the score to 0 to 100, we used the cumulative percentage of the standard normal distribution as the frailty risk score, which was calculated via the Z-scores mentioned before. Then, to ensure that the scores were still in the range of 0 to 100, we propose performance scores of HGS for males and females as Equations (6) and (7) and the performance score of gait speed as (8):

(6) $$P_{HGS\_m}=\int_{-\infty }^{Z_{HGS\_m}}\frac{1}{\sqrt{2\pi }}\exp\left(-\frac{x^{2}}{2}\right)dx$$

(7) $$P_{HGS\_f}=\int_{-\infty }^{Z_{HGS\_f}}\frac{1}{\sqrt{2\pi }}\exp\left(-\frac{x^{2}}{2}\right)dx$$

(8) $$P_{GS}=\int_{-\infty }^{Z_{GS}}\frac{1}{\sqrt{2\pi }}\exp\left(-\frac{x^{2}}{2}\right)dx$$

*P_HGS_m_* and *P_HGS_f_* denote the designed score of the HGS performance of males and females, and *P_GS_* denotes the designed score of the gait speed performance. By following the calculation process described above, we eliminated the sex difference in the HGS distribution. Thus, the scores for males and females had the same distribution and could be discussed together. Finally, to reflect the equal weight given to HGS and gait speed in the J-CHS criteria, we propose a frailty risk score (*P_fr_*) by combining the performance of the two, as expressed by Equation (9).

(9) $$P_{fr}=\left(P_{HGS\_m}+P_{GS}\right)/2\;\mathrm{or}\;P_{fr}=\left(P_{HGS\_f}+P_{GS}\right)/2$$

### 2.7. Evaluation Methods in Model Tests

#### 2.7.1. Test 1

In Test 1, we utilized Bland–Altman (BA) plots [68,69] to assess the limit of agreement (LoA) between IMS-assessed and reference values of gait speed and HGS. We computed both the sample-based LoA and the confidence limits of LoA in the population. To examine the existence of a fixed and proportional bias, we applied a *t*-test and Pearson’s correlation test if the differences and averages between the two methods followed a normal distribution, initially tested by a Kolmogorov–Smirnov (KS) test. The LoA of the 95% confidence interval was established from the perfect agreement (PA) line ± 1.96 × standard deviation (σ), resulting in upper and lower LoAs (ULoA and LLoA). Additionally, the 95% confidence limits of LoA were also determined, which included the upper and lower limits of ULoA (UULoA and LULoA), as well as the upper and lower limits of LLoA (ULLoA and LLLoA). T-tests were used for comparing differences between two groups, and ANOVA was used to compare the differences among three or more groups, with all levels of significance set at *p* < 0.05. 

In the model testing stage, we evaluated the validity of gait speed measurement and HGS estimation based on the ratio of test data in Group II+III, whose BA plots were within the agreement range determined by the model test data for Group I, i.e., the success rate of measurements denoted as *K_A_*. We considered the measurement to be successful by the model when the difference between IMS-measured and reference values was located inside the agreement interval, determined by the data of Group I. We used the optimistic agreement range, i.e., the range between UULoA and LLLoA. Because the test data size was limited, we utilized the probability-distribution-based method [44] to estimate *K_A_* and eliminate randomness. We set the confidence level to 95%, assuming 5% of the measurements as the outliers in this study. If over 95% of data were inside the agreement interval, *K_A_* was considered to be 100%.

In the probability-distribution-based method, we hypothesized that the residual of BA plots for training and test data to the PA line, denoted as *R_A_* and *R_T_*, followed a normal distribution, *R_A_* ~ *N*(*μ_A_*, *σ_A_*^2^) and *R_T_* ~ *N*(*μ_T_*, *σ_T_*^2^). Here, *μ*s and *σ*s mean the averages and standard deviation, respectively. Because the model was based on multivariate regression, theoretically, *μ_A_* ≡ 0. Furthermore, because of the limited data size, we calculated the 95% confidence levels of *μ_T_*, *σ_A_,* and *σ_T_* and obtained their upper and lower limits, (*μ_TL_*, *μ_TU_*), (*σ_AL_*, *σ_AU_*), and (*σ_TL_*, *σ_TU_*), respectively. Hence, if we use an optimistic agreement range, the agreement range of the residual should be fixed as −1.96*σ_AL_* to 1.96*σ_AU_*. By then, *K_A_* should be in the area of *N*(*μ_T_*, *σ_T_*^2^) inside the interval of −1.96*σ_AL_* to 1.96*σ_AU_*. Because *μ_T_* and *σ_T_* are independent of each other, the largest and smallest areas for *N*(*μ_Ti_*, *σ_Ti_*^2^) subject to *μ_Ti_* ∊ [*μ_TL_*, *μ_TU_*] and *σ_Ti_* ∊ [*σ_TL_*, *σ_TU_*] would be the upper and lower limits of *K_A_*, denoted as *K_AU_* and *K_AL_*, which can be expressed by Equations (10) and (11):

(10) $$K_{AU}=\min\left(\max\left(\int_{-1.96\sigma_{AU}}^{1.96\sigma_{AU}}\frac{1}{\sqrt{2\pi \sigma_{Ti}}}\exp\left(-\frac{{\left(x-\mu_{Ti}\right)}^{2}}{2\sigma_{Ti}^{2}}\right)dx\right)/0.95,\;1\right)\;subject\;to\;\mu_{Ti}\in \left[\mu_{TL},\;\mu_{TU}\right],\;\sigma_{Vi}\in \left[\sigma_{TL},\sigma_{TU}\right]$$

(11) $$K_{AL}=\min\left(\min\left(\int_{-1.96\sigma_{AU}}^{1.96\sigma_{AU}}\frac{1}{\sqrt{2\pi \sigma_{Ti}}}\exp\left(-\frac{{\left(x-\mu_{Ti}\right)}^{2}}{2\sigma_{Ti}^{2}}\right)dx\right)/0.95,\;1\right)\;subject\;to\;\mu_{Ti}\in \left[\mu_{TL},\;\mu_{TU}\right],\;\sigma_{Vi}\in \left[\sigma_{TL},\sigma_{TU}\right]$$

#### 2.7.2. Test 2

After calculating the *P_fr_*s of all subjects in Group III, we compared them with the expert-rated scores and calculated the correlation (*r*) between them to evaluate the effectiveness of the designed score. 

For each subject, we obtained a total of six expert-rated scores. We preliminarily tested the reliability of the six expert-rated scores based on the ICC values. The results showed that the ICC(2, 1) was 0.490 (fair), and ICC(2, *k*) was 0.850 (excellent). Moreover, the KS test indicated that the mean score of all subjects corresponding to six raters followed the normal distribution (*p* = 0.987). These results showed that the score indicating a diagnosis of frailty within the next 5 years for the subjects in Group III could be assessed using an average of six expert-rated scores with high reliability. Additionally, as another statistical processing method, we obtained the median values of the six expert-rated scores and the rank of subjects according to each score. For each subject, we then calculated their average rank. Thus, for the other patterns, we used the median value and averaged rank as the reference frailty risk score of the subjects. The correlation analysis between the reference frailty risk score for the other patterns and the designed frailty risk score is shown in the Supplementary Materials.

## 3. Results

### 3.1. SPM Analysis in HGS Estimation Model Construction

**Figure 5:**
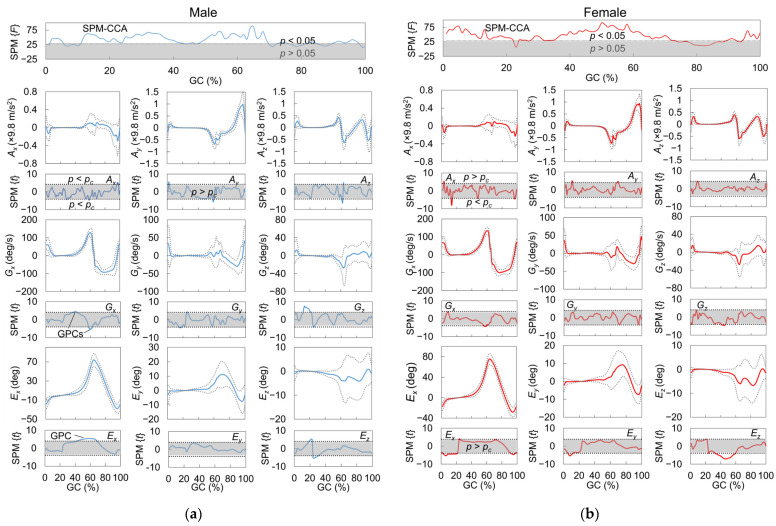
Results of correlation analysis between foot motion and HGS using SPM for both (**a**) males (blue lines) and (**b**) females (red lines). Foot motion waveforms *A_x_*, *A_y_*, *A_z_*, *G_x_*, *G_y_*, and *G_z_* were normalized by the maximum instantaneous speed in one stride. The 95% confidence interval of a waveform is shown by double dotted lines linked to foot motion signals. Statistic curves outside gray zones for each signal type indicate that intervals of GCs significantly correlated with HGS defined as GPCs. GC: gait cycle, SPM{*F*}: *F* statistic of vector field analysis by SPM-CCA, SPM{*t*}: statistic of post hoc scalar trajectory linear correlation test by SPM-PC. Single and double dotted lines linked to SPM{*F*} and SPM{*t*} indicate critical RFT threshold of *F* and Šidák-corrected critical RFT threshold of *t*.

In a comparison between the males and females, their average waveforms appeared approximately similar. In contrast, the standard deviation of waveforms, particularly in the frontal and horizontal plane (*G_y_*, *G_z_*, *E_y_*, and *E_z_*), appeared to have more different shapes (Figure 5).

According to the results of the SPM-CCA, a significant correlation was found between the foot motion signal vectors for most of the stance phase and the end of the swing phase (immediately before HS) and the HGSs for both sexes. A post hoc SPM-PC analysis, represented by statistic SPM{*t*} curves, revealed the strength of the correlation between each type of foot motion signal and the HGS. Significant GC intervals, referred to as GPCs, were identified in the sections of curves that exceeded critical thresholds and correlated with the HGSs. It is worth noting that the GPCs of the acceleration signals were more fragmented due to the smaller smoothness of the acceleration waveform compared to the angular velocities and sole-to-ground Euler angles. The shape of the statistic SPM{*t*} curves and the location of the GPCs varied between males and females (see Figure 5). Consequently, 20 GPCs and 17 GPCs were obtained for males and females, respectively. Filtered by quadricep-activation %GCs (*Q_t_*), 10 GPCs and 14 GPCs ultimately remained for creating the same numbers of IMS predictors.

### 3.2. Feature Selection for HGS Estimation Model

**Figure 6:**
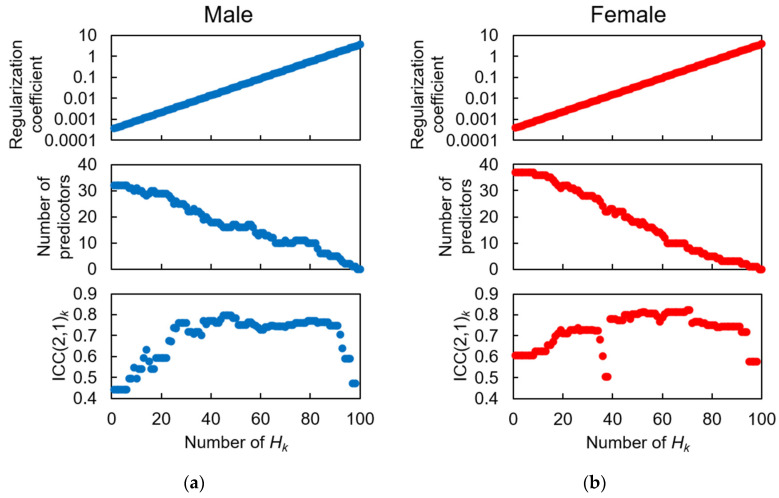
Results of LOSO-LASSO analysis to determine optimal predictor combination, *M_o_*. (**a**) Male, (**b**) female. The upper panels depict the regularization coefficient input into LOSO-LASSO. The middle panels depict the number of predictors output from LOSO-LASSO. The bottom panels depict the ICC(2, 1) values of the models constructed from each predictor combination output from LOSO-LASSO.

To obtain the final optimal predictor combination *M_o_*, consisting of IPA and GP predictors, we inputted a total of 34 and 38 candidate predictors into LOSO-LASSO for males and females, respectively. Referring to Figure 6, we determined *M_o_* for males and females by finding the highest ICC(2, 1), which included 16 and 8 finally selected predictors, respectively. The selected predictors for constructing multivariate linear regression and their correlation analyses with the HGS are listed in Table 3 and Table 4.

Regarding the IPA predictors, age and height were selected for both males and females, with medium to large effect sizes (age: *r* = 0.162 and 0.271; height: *r* = 0.428 and 0.682). In particular, the age for males and height for females had the highest correlation with HGS. These results indicate that the effect of age and body size on HGS was observed. Although the effect size was small (*r* = 0.209), weight was also selected for the estimation model for males. 

Compared to females, more GP predictors were selected for males, with GP16 (*r* = 0.303, medium effect size; *r* = 0.199, small effect size) being present in the predictor list for both sexes. This result suggests that subjects with higher HGSs have lower maximum *G_x_* in the dorsiflexion direction during the swing phase. Except for GP03, which had a medium effect size (*r* = 0.338), the remaining GP predictors (GP05, 08, 09, 10, 18, 19) only had effect sizes classified as none or small. 

**Figure 7:**
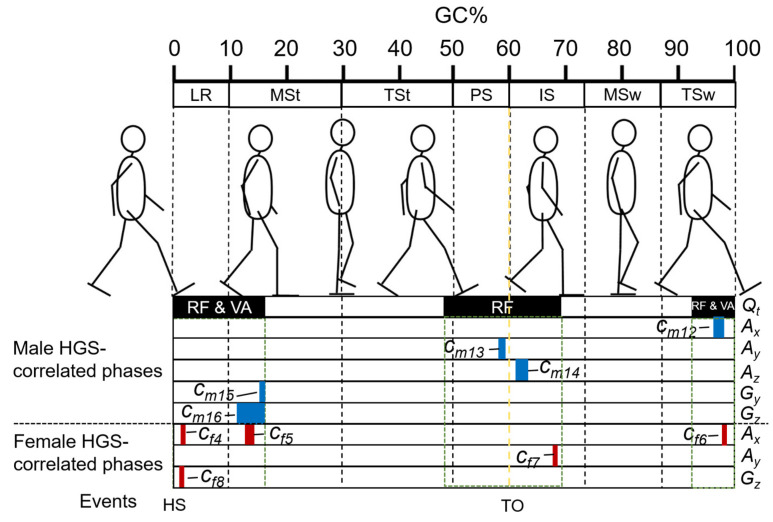
Selected IMS predictors for *M_o_* and their corresponding GPCs for male and female subjects. *Q_t_*’s are marked as black blocks surrounded by green dashed line frames. Selected GPCs of each type of foot motion are also marked as blocks (male: blue, female: red). *Q_t_*: Quadricep-activation %GCs, including %GCs for which only rectus femoris (RF) activated and for both RF and vastus muscles (VAs). LR: loading response; MSt: mid-stance; TSt: terminal stance; PS: pre-swing; IS: initial swing; MSw: mid-swing; TSw: terminal swing; HS: heel strike; TO: toe-off.

For both males and females, five IMS predictors were ultimately selected (*C_m_*_12_–*C_m_*_16_, *C_f_*_4_*–C_f_*_8_) by LOSO-LASSO. The corresponding GPCs are shown in Figure 7. Besides foot motions in the sagittal (*Y-Z*) plane, such as *A_y_* and *A_z_* (*C_m_*_13,14_, *C_f_*_7_), those in the frontal (*X-Z*) and horizontal (*X-Y*) planes, such as *A_x_*, *G_y_*, and *G_z_* (*C_m_*_12,15,16,_ *C_f_*_4–6,8_), were suggested to be essential for HGS estimation. Temporally, major parts of GPCs for females appeared around HS (*C_f_*_4–6,8_), where both the rectus femoris (RF) and vastus muscles (VAs) in the quadriceps were mainly activated. In contrast, besides the GPCs (*C_m_*_12,15,16_) in the %GCs when both the RF and VAs activated, the male subjects also had more GPCs (*C_m_*_13,14_) inside the %GCs for which only the RF activated, which appeared around TO, than the female subjects (*C_f_*_7_). These results may reflect the sex differences in muscle activation patterns during gait. 

By referencing the mean value and linear correlation coefficients of the selected IMS predictors with the HGS, the direction of foot motions during these phases and the changing trend as HGS increased could be determined. Male subjects with stronger HGSs had strong acceleration in the anterior and superior direction (*C_m_*_13,14_) immediately before and after TO. During the early mid-stance phase, when the foot approaches the defined neutral position, male subjects with stronger HGSs had higher angular velocities in the direction of eversion and internal rotation (*C_m_*_15,16_). Immediately after the heel rocker occurred, female subjects with stronger HGSs tended to have lower acceleration in the lateral direction and lower angular velocity in the internal rotation direction (*C_f_*_4,8_). Combining the two predictors, the results may suggest that female subjects with stronger HGSs tend to have a higher ability to land their feet stably and smoothly. After the foot has completely hit the ground, female subjects with higher HGSs tended to have less acceleration in the medial direction (or more acceleration in the lateral direction) (*C_f_*_5_). At the end of the initial swing phase when the lower limb transitioned from acceleration to deceleration, the acceleration in the anterior direction (*C_f_*_7_) of female subjects began to approach zero as HGS increased. 

Furthermore, we also list the coefficients of predictors and their *p*-values in a multivariate regression model in Table 2 and Table 3. Although the linear correlation coefficient with the HGS contained predictors with effect sizes only classified as none or small, the constructed models for both males and females had large effect sizes (*R*^2^ = 0.858, *p* < 0.001, and *R*^2^ = 0.773 and *p* < 0.001, respectively).

### 3.3. Precision Evaluation of Gait Speed, Model Evaluation of HGS Estimation, and Test 1

#### 3.3.1. Gait Speed

**Figure 8:**
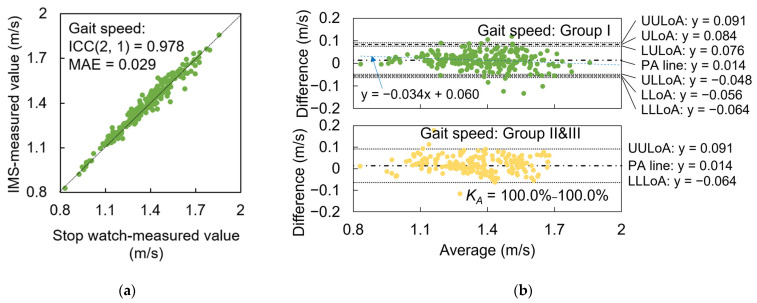
Precision evaluation results of gait speed. (**a**) Agreement plots. (**b**) BA plots of data in Group I (green) and Group II+III (yellow). PA line: black chained line; ULoA and LLoA: black dashed line; UULoA, LULoA, ULLoA, and LLLoA: black dotted line; fitting proportional bias line: blue dashed line. For data in Group II+III, lower to upper limits of *K_A_*, i.e., *K_A_* = *K_AL_* − *K_AU_*, are depicted in the figure.

For all subjects, we evaluated the agreement between the 10 m average gait speed calculated from stopwatch-measured time in one trial and that calculated by averaging all strides of gait speed in 10 m intervals in one trial (see Figure 8a). The ICC agreement reached the “excellent” level with a value of 0.978. Compared to the reference value, the IMS achieved an MAE of 0.029 m/s, which is only 2.1% of the average gait speed of all subjects in Group I. 

From the BA plots of data for Group I (see Figure 8b), we observed a fixed bias indicating that the IMS-measured gait speed was on average 0.014 m/s greater than the stopwatch-measured data (*p* < 0.001). There was also a proportional bias between the two measurements (*r* = −0.173, *p* = 0.006), indicating that IMS slightly overestimated the gait speed when the gait speed became slower (*y* = −0.034*x* + 0.060). The agreement interval for testing data for Group II+III was determined by the BA plots generated from the data for Group I. According to *K_AL_* and *K_AU_* calculated using Equations (8) and (9), the IMS successfully assessed 100% of gait speed data for subjects in Group II+III with an MAE precision of 0.029 m/s.

#### 3.3.2. HGS

**Figure 9:**
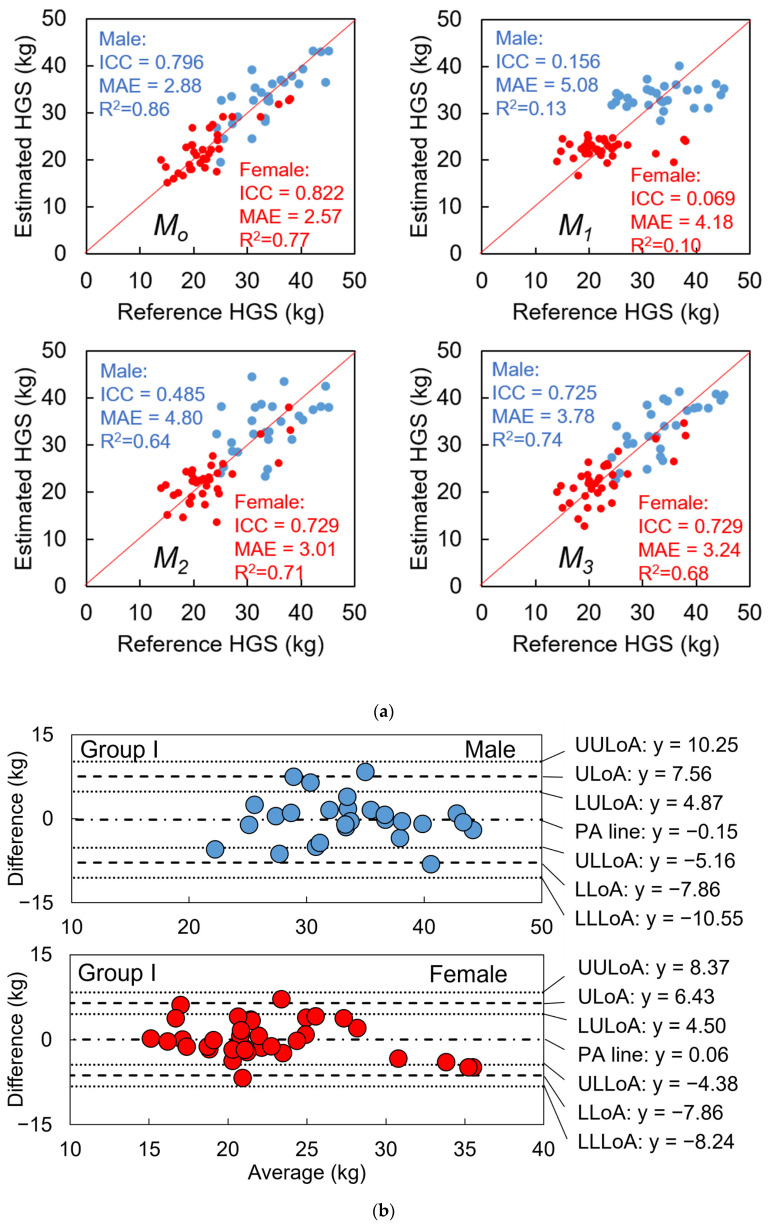

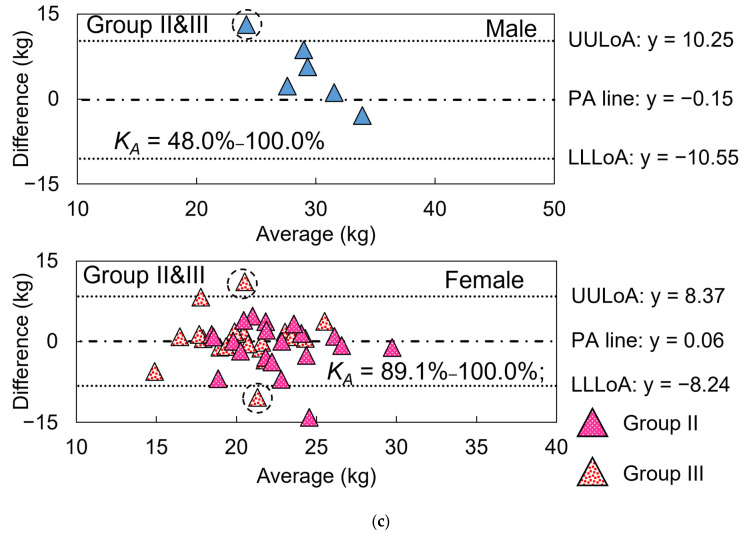
(**a**) HGS estimation agreement plots of males and females by models constructed by predictor combinations of *M*_o_, *M*_1_, *M*_2_, and *M*_3_. Blue and red dots mean data of males and females, and black dashed lines in all panels of (**a**) mean perfect agreement. “ICC” in figures means ICC value of ICC(2, 1). (**b**) Bland–Altman plots of *M*_o_ case for males and females of Group I. PA line: black chained line; ULoA and LLoA: black dashed line; UULoA, LULoA, ULLoA, and LLLoA: black dotted line; fitting proportional bias line: blue dashed line. (**c**) Results of HGS estimation model test using data from Group II+III and optimistic agreement interval determined using data from Group I shown in (**b**). All male subjects belonged to Group III, marked as blue triangles. Lower to upper limits of *K_A_*, i.e., *K_A_* = *K_AL_* − *K_AU_*, are depicted in (**c**). Black dashed circle in (**c**) means subjects in Group III who did not agree with the reference data well.

The results presented in Figure 9a suggest that gait speed alone or combined with other common GPs is not an effective predictor for estimating HGS. From the results shown in Figure 9a, it can be inferred that gait speed is significantly correlated with HGS among male and female subjects, with moderate effect sizes (*r* = 0.384, 0.337, *p* = 0.048, 0.048), but estimating HGS based solely on gait speed is not feasible due to the poor ICC agreement between the estimated and reference values. However, when additional GPs were added as predictors by using the LOSO-LASSO model (*M*_2_), significant improvements were observed for ICC, MAE, and *R*^2^. The ICC agreement for males and females improved from poor to fair and good, respectively, while the *R*^2^ improved from small to large. Specifically, for the males, the ICC agreement improved from fair to good with the aid of IPAs. Additionally, the optimal model (*M_o_*) that included IMS predictors resulted in a substantial improvement in ICC agreements, MAE, and *R*^2^, where the ICCs reached excellent for both males and females with MAE and *R*^2^ values improving to 2.88 and 2.57 kg and 0.86 and 0.77, respectively. Further details on *M*_2_ and *M*_3_ predictor combinations can be found in the Supplementary Materials.

The differences between the reference and estimated values of Group I data followed a normal distribution, as shown in Figure 9b. The Bland–Altman plots of Group I for both males and females did not reveal any proportional biases (*p* = 0.76, 0.09) between the measurements. In terms of the HGS model test results using Group II+III data, the HGS estimation was successful for 5/6 males and 36/39 females within the agreement interval. According to Equations (10) and (11), 48.0–100.0% of male subjects and 89.1–100.0% of female subjects were estimated successfully. However, it appeared that HGS was overestimated for males in Group II+III.

### 3.4. Test 2: Validity of Designed Frailty Risk Score with Estimated HGS

In Test 2, the scores of male and female subjects were evaluated together because the experts did not consider biological sex. For both males and females in Group III, there was no significant linear correlation between HGS and gait speed (*r* = 0.025, *p* = 0.963, and *r* = 0.170, *p* = 0.302, respectively). Even after calculating *P_HGS_* and *P_GS_*, there was still no significant linear correlation between the performance scores (*r* = 0.363, *p* = 0.075), possibly due to the small sample size and insufficient statistical power. 

**Figure 10:**
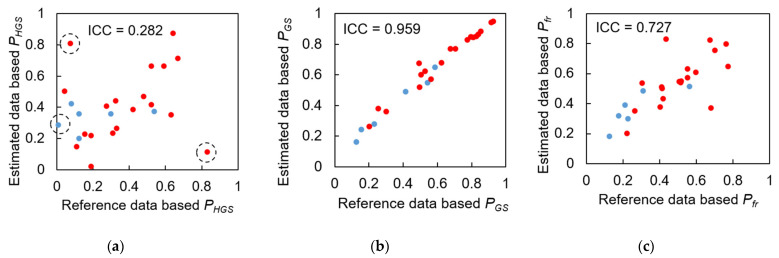
ICC agreement between three types of performance scores calculated from reference and IMS-estimated values: (**a**) *P_HGS_*, (**b**) *P_GS_*, (**c**) *P_fr_*. Points in dashed circles mean subjects whose data are outside the agreement interval in Figure 9c (the same data in dashed circles in Figure 9c). Blue points: male subjects. Red points: female subjects.

The ICC agreement between the three types of performance scores based on reference and IMS-estimated values is shown in Figure 10. *P_GS_* had an excellent level of agreement with an ICC(2,1) of 0.959 (Figure 10b), while *P_HGS_* only had a poor level with an ICC(2,1) of 0.282 (Figure 10a), possibly due to a few subjects who did not agree well with the reference data. However, when *P_HGS_* and *P_GS_* were combined with *P_fr_*, the ICC value improved to a good level at 0.727 (Figure 10c). 

**Figure 11:**
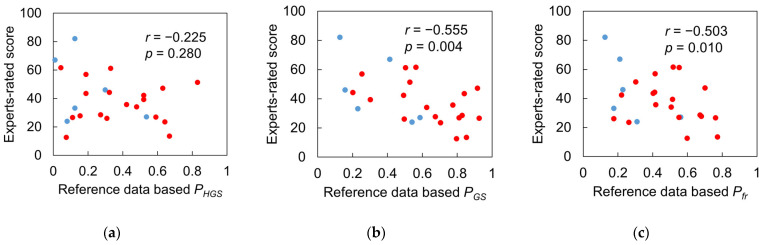
Correlations between expert-rated score and three types of performance scores calculated from reference value: (**a**) *P_HGS_*, (**b**) *P_GS_*, (**c**) *P_fr_*. Blue points: male subjects. Red points: female subjects.

**Figure 12:**
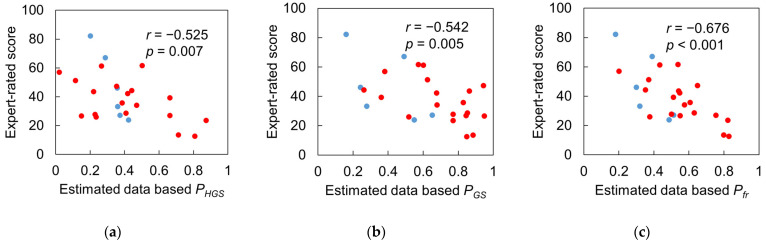
Correlations between expert-rated score and three types of performance scores calculated from IMS-estimated value: (**a**) *P_HGS_*, (**b**) *P_GS_*, (**c**) *P_fr_*. Blue points: male subjects. Red points: female subjects.

Figure 11 and Figure 12 show the correlations between the expert-rated score and the three types of performance scores based on reference and IMS-estimated values. The expert-rated score had a significant negative correlation with reference data-based *P_GS_* and *P_fr_*, with large effect sizes (*r* = −0.555, −0.503; *p* = 0.004, 0.010), but not with reference data-based *P_HGS_* (*r* = −0.225, *p* = 0.280) (Figure 11). However, the *P_HGS_* based on IMS-estimated data had a significant negative correlation with the expert-rated score with a large effect size (*r* = −0.525, *p* = 0.007), and the *P_fr_* based on IMS-estimated data had a higher effect size (*r* = −0.676, *p* < 0.001) than the reference data-based one. These results indicate that the performance scores based on IMS-estimated data are more consistent with the experts’ diagnostic reasoning.

**Figure 13:**
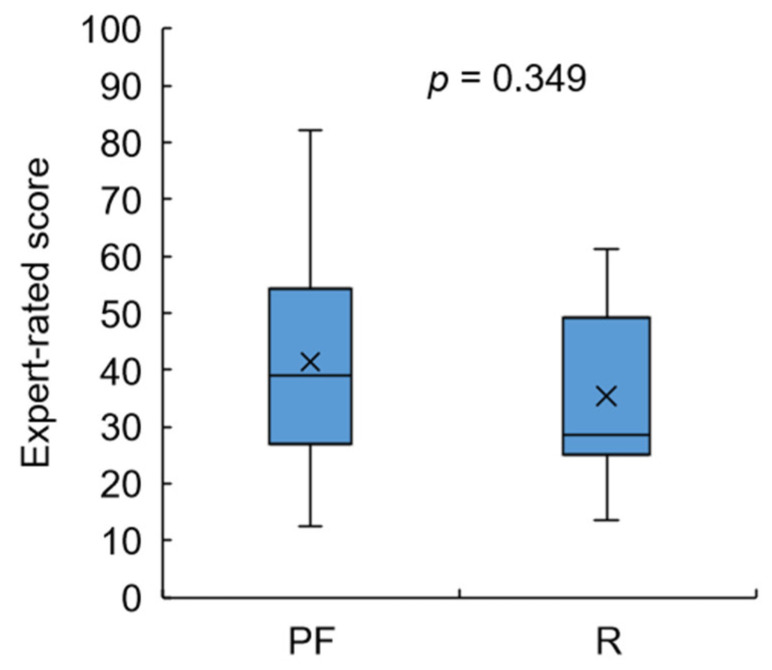
Boxplot of expert-rated score in pre-frail and robust groups. Lines in the boxes indicate the median values; crosses in the boxes indicate the mean values of each group. PF: pre-frail, R: robust.

We conducted a statistical analysis of the difference in expert-rated scores between subjects classified as pre-frail and robust based on the J-CHS score (Figure 13). We found no significant difference between the subject groups that scored 1 to 2 and 0, which may be due to the difficulty in precisely scoring subjects who are on the boundary of robust/pre-frail conditions based only on gait observation. Nevertheless, the average value of the robust group was lower than that of the pre-frail group. 

**Figure 14:**
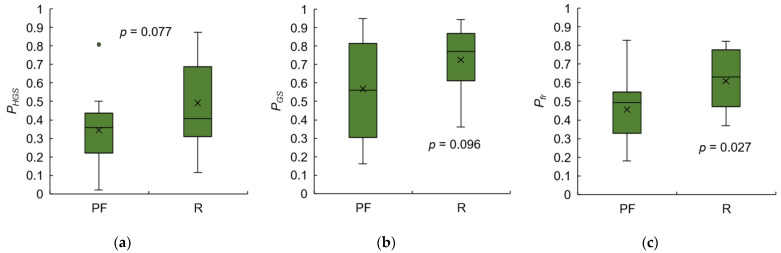
Boxplot of three types of performance scores calculated from IMS-estimated values in pre-frail and robust groups: (**a**) *P_HGS_*, (**b**) *P_GS_*, (**c**) *P_fr_*. The green dot in (**a**) means the outlier point (values exceeding 1.5 times the interquartile range are displayed as outliers). Lines in the boxes indicate the median values; crosses in the boxes indicate the mean values of each group. PF: pre-frail, R: robust.

Furthermore, we tested the three types of performance scores on the basis of IMS-estimated data for the pre-frail and robust groups (Figure 14). Despite the *t*-test showing no significant difference between the two groups in either HGS or gait speed performance scores, the overall performance *P_fr_* of the robust group was significantly higher than that of the pre-frail group. This suggests that the frailty risk score was consistent with the J-CHS criteria.

## 4. Discussion

### 4.1. Some Significant GP Predictors for HGS Estimation

Although gait speed has been suggested to be correlated with HGS in previous studies [32,33], in this study, gait speed was not selected as a predictor for the HGS estimation model for either male or female subjects. Instead, other spatiotemporal parameters were discovered to be significant for HGS estimation in our designed model. These parameters played a key role in the optimal model for HGS estimation based on the analysis of ICC agreement. After applying the LOSO-LASSO method, essential GP predictors were selected. 

One of the essential GP predictors is the maximum sole-to-ground angle in the dorsiflexion direction (GP03), which has a relatively high positive correlation with HGS in males. As shown in Figure 5, GP03 occurs immediately before heel strike. During this phase of the gait cycle, the ankle joint is in a neutral status; i.e., the foot is perpendicular to the tibia. Therefore, the value of GP03 is determined by the degree of knee extension [70]. When the knee extensor, i.e., quadriceps, becomes weaker, the knee cannot be extended sufficiently, which causes GP03 to become smaller. 

**Figure 15:**
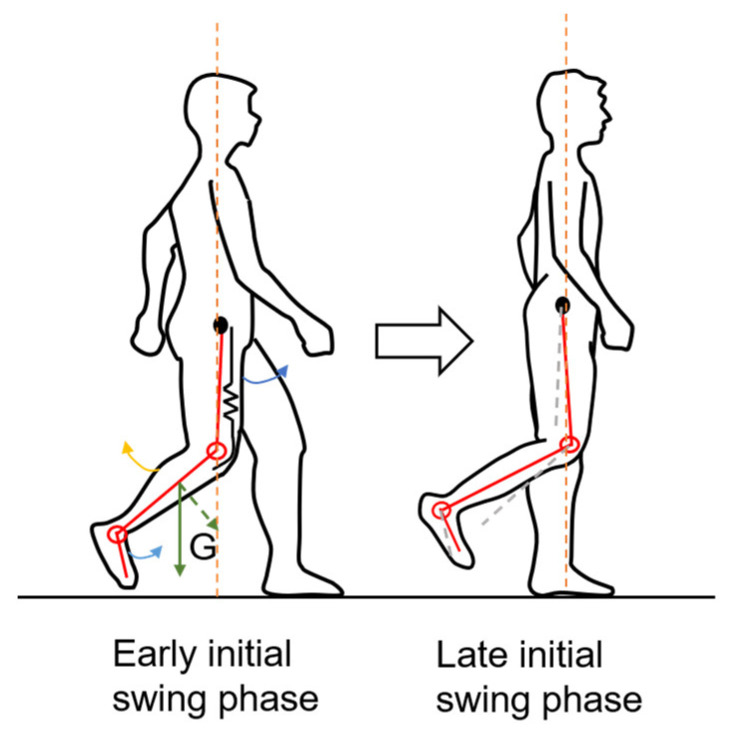
Gait motion of early and late initial swing phase. Spring mark means rectus femoris. Red lines mean segments of lower limbs. Gray dashed line means original position of each segment. Red circles mean approximate position of knee and ankle joints. Orange dashed line means central line of body. Black bold point means approximate position of hip joint. Blue arrow means rotational motion direction, which increases angular velocity in dorsiflexion direction on IMS. Yellow arrow means rotational motion direction, which decreases angular velocity in dorsiflexion direction on IMS. Green line arrow means direction of gravity, and green dashed arrow means projection of gravity vector in direction perpendicular to segment of lower leg.

Another essential GP predictor for both sexes is the maximum angular velocity in the dorsiflexion direction during the swing phase (GP16). Unlike GP03, the negative correlation coefficient between HGS and GP16 suggests that subjects with a higher HGS have a lower absolute value of GP16, i.e., a value closer to zero. This can be explained as follows: According to Figure 5, GP16 is most likely to occur during the initial swing phase. During this phase, the upper leg rotates forward (blue arrow on upper leg in Figure 15), and the knee joint gradually increases flexion. Passively, the lower leg lifts behind the central line of the body (yellow arrow in Figure 15), which prevents the lower leg from rotating forward too early by overcoming the gravity force (green arrow in Figure 15). At the same time, the ankle joint spontaneously reduces plantarflexion, which rotates the foot forward (blue arrow on foot in Figure 15). The *G_x_* waveform during this phase reflects the counterbalance motion of the knee and ankle joint [70]. Furthermore, Nene et al. [71] suggested that the rectus femoris muscle controls the degree of knee flexion. Therefore, when the quadriceps, especially the rectus femoris, becomes weaker, the antagonizing muscle power that prevents the lower leg from rotating forward along with gravity also decreases. Consequently, GP16 becomes larger in the dorsiflexion direction.

### 4.2. Some Significant IMS Predictors for HGS Estimation

Through SPM analysis of the correlation between HGS and the foot motion waveforms, we discovered a number of effective IMS predictors, and five IMS predictors were finally selected by LOSO-LASSO. As shown in Figure 7, the major parts of GPCs for the females appeared around HS (*C_f_*_4–6,8_), where both the RF and VAs in the quadriceps were mainly activated, while the male subjects also had more GPCs (*C_m_*_13,14_) inside the %GCs for which only the RF was activated, which appeared around TO, than the female subjects (*C_f_*_7_). Di Nardo et al. [72] suggested that female subjects have more complex activation patterns in VAs. Bailey et al. [73] indicated that in older adults’ gait, the activation level of RF for males is higher than that for females according to a study using electromyography. The results shown in Figure 6 may reflect sex-dependent muscle activation during gait.

Kobayashi et al. [48] demonstrated the differences in GPs between sexes. Rowe et al. [74] analyzed the sex differences in kinetics and kinematics of lower limbs in detail, which indicated that more differences were found in frontal and horizontal planes. In this study, we also observed a difference in foot motion waveforms, which also belong to the gait in the lower limbs, between male and female subjects, especially in the frontal plane and transverse plane. Our results agreed with the findings demonstrated in these previous studies.

We analyzed the correlation between the balance ability, represented by the outcome of the FRT, and foot motion with the same subjects in Group I in our previous study [45]. We found several significant GPCs by paying attention to the gait phases related to the activation of the tibialis anterior (TA) and calf muscles (gastrocnemius (GA) and soleus (SO)). The TA has two periods of activity: one is during the early stance phase (1–15%GC), and the other is from the late pre-swing to the end swing phase (55–100%GC). Partial quadricep-activated gait phases overlap with the TA at the moments before and after HS. Different from the GPCs in the HGS assessment model, there were no GPCs selected at the end of the swing phase, i.e., the second period of TA activity, in the balance ability assessment model, which may suggest that the power needed for knee extension contributes less to balance ability. In contrast, similar to the balance ability assessment model, the HGS estimation model also has GPCs in the early stance phase (the first period of TA activity). In this period, the quadriceps control the lower limb to prevent excessive knee flexion, and at the same time, the TA contributes to decelerating the passive plantarflexion and foot pronation to make the posture more stable [69]. A previous study discovered that HGS was significantly correlated with the outcome of FRT in the older Asian population [75]. We also found that the HGS was significantly correlated with the outcome of FRT in our study (male: *r* = 0.456, *p* = 0.017; female: *r* = 0.390, *p* = 0.020). We think that the correlation may be related to the common parts of GPCs in both models during the early stance phase right after HS.

### 4.3. Results Regarding Model Test and Designed Frailty Risk Score

The agreement between reference data and IMS-estimated data for *P_HGS_* only reached a “poor” level due to the estimated HGSs of one male and two female subjects that deviated from the reference values (Figure 10a, marked with three dashed black circles). It appears that IMS did not estimate the HGS of these three subjects accurately compared to the hydraulic hand dynamometer which is considered as the gold standard. However, the reference HGS only reflected the static systemic muscle strength of the upper limb. Figure 11a indicates that there was no significant correlation between the reference HGS and the expert-rated score, while Figure 12a suggests that IMS-estimated HGS was significantly correlated with the expert-rated score. Furthermore, compared to the results in Figure 11c and Figure 12c, our designed frailty risk score using IMS-estimated values agreed more with the clinical experts. These results may be due to the fact that our model focused on gait performance and reflected dynamic muscle conditions via the lower limbs. Moreover, experienced clinicians and physiotherapists tend to rely on information extracted from gait observation for making their decisions in clinical practice. The ICC(2, *k*) of the HGS between reference and IMS-estimated values reached 0.886 and 0.902 for males and females, respectively, indicating that the average value of the dynamometer and IMS-estimated HGS can be used in clinical practice to better approach subjects’ systemic muscle strength reality. 

Our designed frailty risk score was significantly correlated with the expert-rated score (*r* = −0.676, *p* < 0.001), indicating the reliability of frailty risk assessment using IMS and our designed frailty risk score. Additionally, significant differences were found in the designed frailty risk score between subjects in the group with a J-CHS score of 0 and those with a score of 1 to 2, further supporting the use of our proposed method for frailty assessment.

### 4.4. Outlook for this Technology

As a feasibility study, we temporarily recorded foot motion data in the onboard memory of IMSs during the experiments and transferred the data to a personal computer after the gait measurements were completed. However, in daily use, a real-time algorithm for frailty assessment is necessary. In our previous studies, we proposed an online algorithm for estimating stride parameters for daily gait analysis using an IMS [29], as well as an algorithm for integrating the process of IMS predictor construction into the online algorithm [44]. By using the same algorithms, we believe that daily frailty assessment can be performed using an IMS. 

In this study, we did not diagnose whether the subjects were frail or not, as all recruited subjects were able to come to the laboratory using public transportation. Therefore, we assumed that they were in generally good health. The characteristics of the subjects can be observed from their J-CHS scores, but the frailty risk score does not directly represent the probability of an individual being diagnosed as frail. Instead, it reflects the relative degree of frailty in the population. To obtain more evidence supporting our frailty risk score, future longitudinal cohort studies should be conducted to track the frailty of subjects. Additionally, an epidemiological study regarding the frailty risk score is needed to improve its interpretability in connection with the real probability of being diagnosed as frail. 

To improve the HGS estimation model’s precision, future studies should focus on increasing the sample size. To improve the agreement of the frailty risk score with experts’ diagnostic reasoning, IMS estimation should include three additional items in the J-CHS criteria: activity level, fatigue, and weight loss. Gokalgandhi et al. [28] suggested that daily activity and calorie consumption could be monitored by smart shoes. However, an estimation method via IMSs for the other two items is still lacking. In their study, Luo et al. [76] proposed a pilot method for assessing fatigue via wearable sensors that utilized vital signs such as heart rate, blood pressure, skin temperature, and steps, but did not include other GPs. Previous kinematic studies [77,78] have shown that fatigue and weight loss can impact kinematic patterns. Therefore, assessing fatigue and weight loss using IMSs alone is promising but requires further investigation in the future.

## 5. Conclusions

In this study, we demonstrated the potential for long-term frailty assessment using IMSs, which required two key tasks. The first task was to accurately measure gait speed using IMSs and construct an HGS estimation model via foot motion. The second task was to create a frailty risk score that can continuously assess frailty and validate its effectiveness. 

For the first task, we confirmed that IMSs can measure gait speed with high accuracy, with an ICC agreement with reference data of over 0.97. By analyzing the correlation between HGS and foot motion waveforms using SPM-LOSO-LASSO, we discovered novel GPs and IMS predictors for HGS estimation. Specifically, we found that male subjects had more GPC components inside the %GCs for which only the RF was activated, while female subjects had more GPC components inside the %GCs for which both the VAs and RF were activated. We successfully constructed sex-dependent HGS estimation models, both of which achieved “excellent” ICC agreement, MAEs below 2.9 kg, and large effect sizes (*R*^2^ over 0.77). By testing the model on a separate sample of subjects, we found that 48.0–100% of males and 89.1–100% of females were within the agreement interval, indicating the robustness of our model for other older individuals. 

For the second task, we successfully designed a novel analog frailty risk score by combining the HGS performance and gait speed performance of the subjects aiding by the normal distribution of HGS and gait speed of the Asian older population. This score had a large effect size correlation with the expert-rated score, demonstrating its validity and agreement with clinical experts’ diagnostic reasoning. 

In the future, an epidemiological study is needed to improve the interpretability of the frailty risk score in connection with the real probability of being diagnosed with frailty. Furthermore, to better align with clinical experts’ diagnostic reasoning, an IMS assessment of three other items related to activity, weight loss, and fatigue is needed. 

## Figures and Tables

**Table 1:** Demographic data and characteristics of subjects. Subjects for model construction (Group I), Test 1 (Group II+III), and Test 2 are summarized.

		Overall Mean ± SD (Min–Max)	Male Mean ± SD (Min–Max)	Female Mean ± SD (Min–Max)
Group I	Number	62	27	35
	Data size	248	108	140
	Age (years)	70.6 ± 6.8 (60.0–84.0)	70.3 ± 7.7 (60.0–84.0)	70.9 ± 5.9 (60.0–82.0)
	Height (cm)	160.0 ± 8.2 (140.0–176.0)	166.7 ± 4.2 (160.0–176.0)	154.9 ± 6.6 (140.0–171.0)
	Weight (kg)	59.9 ± 11.0 (37.0–89.0)	66.8 ± 8.8 (53.0–89.0)	54.7 ± 9.4 (37.0–80.0)
	BMI	23.3 ± 3.1 (15.2–32.9)	24.0 ± 2.6 (19.2–29.4)	22.8 ± 3.4 (15.2–32.9)
	HGS (kg)	27.4 ± 8.1 (14.0–45.2)	33.7 ± 6.1 (24.3–45.2)	22.6 ± 5.8 (14.0–38.0)
	Gait speed (m/s)	1.37 ± 0.18 (0.91–1.83)	1.35 ± 0.20 (0.99–1.83)	1.39 ± 0.17 (0.91–1.72)
Group II+III	Number	45	6	39
	Data size	180	24	156
	Age (years)	71.1 ± 7.1 (50.0–86.0)	77.7 ± 5.4 (70.0–86.0)	70.1 ± 6.8 (50.0–83.0)
	Height (cm)	155.3 ± 6.1 (146.0–172.0)	166.5 ± 4.8 (160.0–172.0)	153.6 ± 4.0 (146.0–164.5)
	Weight (kg)	53.2 ± 10.1 (34.0–76.0)	63.1 ± 12.2 (41.0–76.0)	51.7 ± 8.8 (34.0–73.0)
	BMI	22.0 ± 3.6 (14.5–31.1)	22.7 ± 3.8 (15.2–26.0)	21.9 ± 3.6 (14.5–31.1)
	HGS (kg)	22.3 ± 4.5 (13.7–35.4)	26.9 ± 5.6 (17.6–35.4)	21.6 ± 3.9 (13.7–31.6)
	Gait speed (m/s)	1.33 ± 0.19 (0.75–1.64)	1.18 ± 0.14 (1.02–1.34)	1.35 ± 0.19 (0.75–1.64)
Group III	Number	25	6	19
	Data size	100	24	76
	Age (years)	75.1 ± 5.8 (65.0–86.0)	77.7 ± 5.4 (70.0–86.0)	74.2 ± 5.8 (65.0–83.0)
	Height (cm)	156.4 ± 6.6 (146.0–172.0)	166.5 ± 4.8 (160.0–172.0)	153.4 ± 3.0 (146.0–160.0)
	Weight (kg)	51.4 ± 10.9 (34.0–76.0)	63.1 ± 12.2 (41.0–76.0)	47.9 ± 7.5 (34.0–62.0)
	BMI	20.9 ± 3.6 (14.5–27.7)	22.7 ± 3.8 (15.2–26.0)	20.4 ± 3.4 (14.5–27.7)
	HGS (kg)	21.9 ± 4.9 (13.7–35.4)	26.9 ± 5.6 (17.6–35.4)	20.1 ± 3.4 (13.7–26.5)
	Gait speed (m/s)	1.33 ± 0.18 (1.02–1.64)	1.18 ± 0.14 (1.02–1.34)	1.39 ± 0.16 (1.09–1.64)
	J-CHS score: 0 (Robust)	10	1	9
	J-CHS score: 1–2 (Pre-frail)	15	5	10
	J-CHS score: >2 (Frail)	0	0	0
	Average expert-rated score	39.3 ± 17.1 (12.6–82.2)	46.6 ± 23.5 (23.9–82.2)	37.0 ± 14.6 (12.6–61.6)

SD: standard deviation. HGS and gait speed are reference values.

**Table 2:** The 20 types of GPs designed for model construction.

No.	Description	Unit
GP01	Stride length	m
GP02	One-stride gait velocity	m/s
GP03	Maximum *E_x_* in dorsiflexion direction	deg
GP04	Maximum *E_x_* in plantarflexion direction	deg
GP05	Maximum circumduction	m
GP06	Maximum foot height	m
GP07	Toe in/out angle	deg
GP08	*E_y_* at HS	deg
GP09	*E_y_* at TO	deg
GP10	Cadence	step/min
GP11	Stance phase time	s
GP12	Swing phase time	s
GP13	Double support time 1 (loading response)	s
GP14	Double support time 2 (pre-swing)	s
GP15	Maximum *G_x_* in plantarflexion direction during swing phase	deg/s
GP16	Maximum *G_x_* in dorsiflexion direction during swing phase	deg/s
GP17	Maximum instantaneous velocity in one stride	m/s
GP18	Maximum *A_z_* in superior direction during swing phase	9.8 m/s^2^
GP19	Duration of HS to foot flat	s
GP20	Duration of foot flat	s

GP: gait parameter. GP01-GP07 were calculated using the method of Fukushi et al. [29]. GP13, GP14, GP19, and GP20 were calculated using the method of Huang et al. [62]. Deg: degree.

**Table 3:** Predictors in constructed multivariate linear regression model and their correlation analyses with HGS for males.

No.	Detail	Mean (SD)	*r*	Coef.	*p_m_*
Int.	Interception			37.9	0.050
*C*_*m*1_	Age	70.3 (7.7)	−0.599	−0.236	0.000
*C*_*m*2_	Height	166.7 (4.2)	0.428	0.185	0.055
*C*_*m*3_	Weight	66.8 (8.8)	0.209	0.191	0.000
*C*_*m*4_	GP03	31.64 (4.83)	0.338	−0.525	0.000
*C*_*m*5_	GP05	1.88 × 10^−2^ (0.75 × 10^−2^)	0.204	132	0.006
*C*_*m*6_	GP08	−5.21 (3.83)	−0.005	0.262	0.009
*C*_*m*7_	GP09	3.64 (3.88)	−0.049	0.246	0.013
*C*_*m*8_	GP10	112.28 (9.20)	0.052	−0.222	0.000
*C*_*m*9_	GP16	−97.05 (5.46)	0.303	0.314	0.000
*C*_*m*10_	GP18	6.35 × 10^−1^ (0.61 × 10^−1^)	−0.190	15.9	0.017
*C*_*m*11_	GP19	9.41 × 10^−2^ (2.13 × 10^−2^)	0.065	116	0.000
*C*_*m*12_	*A_x_*, 97 to 98	−2.67 × 10^−1^ (1.41 × 10^−1^)	0.462	11.2	0.001
*C*_*m*13_	*A_y_*, 59	−5.76 × 10^−1^ (1.04 × 10^−1^)	−0.487	−26.0	0.000
*C*_*m*14_	*A_z_*, 61 to 63	−3.43 × 10^−1^ (0.84 × 10^−1^)	−0.389	−8.11	0.054
*C*_*m*15_	*G_y_*, 15 to 16	−5.26 × 10^−1^ (8.01 × 10^−1^)	−0.387	−1.58	0.000
*C*_*m*16_	*G_z_*, 12 to 16	4.35 × 10^−1^ (3.75 × 10^−1^)	0.582	4.98	0.000

GP03: maximum *E_x_* in dorsiflexion direction; GP05: maximum circumduction; GP09: *E_y_* at TO; GP10: cadence; GP16: maximum *G_x_* in dorsiflexion direction during swing phase; GP18: maximum *A_z_* in superior direction during swing phase; GP19: Duration of HS to foot flat. GP05 was normalized by height of subject; GP16, GP18, and GP19 were all normalized by maximum instantaneous speed in one stride. *C*_*m*12_ to *C*_*m*16_: IMS predictors, signal type, and interval range of GPCs are depicted in “Detail” column. Interval range is in %GC. Units of IMS predictors were the same as signals. *C*_*m*12_ to *C*_*m*16_ were all normalized by maximum instantaneous speed in one stride. SD: standard deviation, *r*: linear correlation coefficient of predictor with HGS; Coef.: coefficient of multivariate regression model using all participants’ data; *p_m_*: *p*-value of coefficient of multivariate regression model, with significance level of *p_m_* < 0.05.

**Table 4:** Predictors in constructed multivariate linear regression model and their correlation analyses with HGS for females.

No.	Detail	Mean (SD)	*r*	Coef.	*p_m_*
Int.	Interception			−17.3	0.178
*C*_*f*1_	Age	70.9 (5.9)	−0.517	−0.349	0.000
*C*_*f*2_	Height	154.9 (6.6)	0.682	0.374	0.000
*C*_*f*3_	GP16	−102.37 (7.67)	0.199	−0.095	0.025
*C*_*f*4_	*A_x_*, 3	−1.31 × 10^−1^ (0.76 × 10^−1^)	0.419	28.9	0.000
*C*_*f*5_	*A_x_*, 13 to 14	−2.43 × 10^−3^ (2.43 × 10^−3^)	−0.529	−819	0.000
*C*_*f*6_	*A_x_*, 97	−2.25 × 10^−1^ (1.30 × 10^−1^)	−0.362	−7.03	0.002
*C*_*f*7_	*A_y_*, 69	−2.81 × 10^−1^ (0.46 × 10^−2^)	0.374	22.5	0.000
*C*_*f*8_	*G_z_*, 2	14.44 (9.76)	−0.325	0.244	0.000

GP16: maximum *G_x_* in dorsiflexion direction during swing phase, which was normalized by maximum instantaneous speed in one stride. *C*_*f*4_ to *C*_*f*8_: IMS predictors, signal type, and interval range of GPCs are depicted in “Detail” column. Interval range is in %GC. *C_f_*_4_ to *C_f_*_8_ were all normalized by maximum instantaneous speed. Units of IMS predictors were the same as signals. SD: standard deviation; *r*: linear correlation coefficient of predictor with HGS, Coef.: coefficient of multivariate regression model using all participants’ data, *p_m_*: *p*-value of coefficient of multivariate regression model, with significance level of *p_m_* < 0.05.

## Data Availability

Data are unavailable due to privacy or ethical restrictions.

## Supplementary Materials

The following supporting information can be downloaded at: https://www.mdpi.com/article/10.3390/s23125446/s1. Figure S1. Results of LOSO-LASSO analysis to determine (a) *M_2_* and (b) *M_3_*; Table S1. Optimal predictor combination in *M_2_* and *M_3_*; Figure S2. Correlations between expert-rated median score and three types of performance scores calculated from IMS-estimated value: (a) *P_HGS_*, (b) *P_GS_*, (c) *P_fr_*. Figure S3. Correlations between expert-rated mean rank and three types of performance scores calculated from IMS-estimated value: (a) *P_HGS_*, (b) *P_GS_*, (c) *P_fr_*.

## Appendix A

The acronyms and symbols used in this manuscript are listed in Table A1.

**Table A1:** The acronyms and symbols used in the manuscript.

Symbol	Description	Symbol	Description
%GC	Percentage gait cycle	LR	Loading response
AWGS	Asian Working Group on Sarcopenia	LULoA	Lower limit of ULoA
*A_x_*	The acceleration signal of *x*-axis	*M*_1_–*M*_3_	Three other patterns of predictor combinations
*A_y_*	The acceleration signal of *y*-axis	MAE	Mean absolute error
*A_z_*	The acceleration signal of *z*-axis	MCU	Micro-control unit
BA plots	Bland–Altman plots	*M_o_*	The optimal model
BMI	Body mass index	MSt	Mid-stance
CCA	Canonical correlation analysis	MSw	Mid-swing
CHS	Cardiovascular health study	PeC	Pearson’s correlation
*C_i_*	The i-th predictor variable	*P_fr_*	Frailty risk score
EMBC	Conference of the IEEE Engineering in Medicine and Biology Society	PGS	Designed score of the gait speed performance
*E_x_*	The SGA signal of *x*-axis	*P_HGS_f_*	Designed score of the HGS performance of females
*E_y_*	The SGA signal of *y*-axis	*P_HGS_m_*	Designed score of the HGS performance of males
*E_z_*	The SGA signal of *z*-axis	PS	Pre-swing
FRT	Functional reach test	*Q_t_*	Quadricep-activation %GCs
GA	Gastrocnemius	*r*	Pearson’s coefficient of correlation
GC	Gait cycle	*R* ^2^	Adjusted coefficient of determination
GP	Gait parameter	*R_A_*	Residual of BA plots for training data
GPC	Gait phase cluster	*R_T_*	Residual of BA plots for test data
GS	Gait speed	RF	Rectus femoris
G_x_	The angular velocity signal of x-axis	RFT	Random field theory
G*_y_*	The angular velocity signal of *y*-axis	RTC	Real-time clock
G*_z_*	The angular velocity signal of *z*-axis	SGA	Sole-to-ground angle
*H*_1_–*H*_100_	100 candidate models	SO	Soleus
HGS	Hand grip strength	SPM	Statistical parametric mapping
*HGS_f_*	HGS of the female subjects	SPM{*F*}	*F* statistic of vector field analysis by SPM-CCA
*HGS_m_*	HGS of the male subjects	SPM{*t*}	Statistic of post hoc scalar trajectory linear correlation test by SPM-PC
HS	Heel strike	TA	Tibialis anterior
ICC	Intraclass correlation coefficient	*T_e_*	The end of %GCs of GPCs
IMS	In-shoe motion sensor	TO	Toe-off
IMU	Inertial measurement unit	TSt	Terminal stance
IPA	Individual physical attribute	*T_s_*	The start of %GCs of GPCs
ISw	Initial swing	TSw	Terminal swing
J-CHS	Japanese version of the Cardiovascular Health Study	ULLoA	Upper limit of LLoA
*K_A_*	The success rate of measurements	ULoA	Upper LoA
*K_AL_*	Lower limit of KA	UULoA	Upper limit of ULoA
*K_AU_*	Upper limit of KA	VA	Vastus muscle
KS test	Kolmogorov–Smirnov test	*W*	The waveform of the foot motion signals
LASSO	Least absolute shrinkage and selection operator	*Z_GS_*	Z-scores of the gait speed
LLLoA	Lower limit of LLoA	*Z_HGS_m_*	Z-scores of the HGS performance of males
LLoA	Lower LoA	*Z_HGS_f_*	Z-scores of the HGS performance of females
LoA	Limit of agreement	*β*	The set of fitted least-squares regression coefficients
LOSO	Leave-one-subject-out	*β*_0_	The residual of the linear regression
LOSOCV	Leave-one-subject-out cross-validation	*λ*	Non-negative regularization parameter input to LASSO
