# Supplementary material for: Healthcare Application of In-Shoe Motion Sensor for Older Adults: Frailty Assessment Using Foot Motion during Gait
Source: Sensors (Basel). 2023 Jun 8;23(12):5446. doi: 10.3390/s23125446 (PMC10304868; doi:10.3390/s23125446)
Supplement: Supplementary file 1 [file sensors-23-05446-s001.zip › sensors-2330650-supplementary.pdf]

sensors

MDPI

Supplementary materials

1. Details on selected predictors for M_2 and M_3

In this study, to construct a hand grip strength (HGS) estimation model from foot motion by using an in-shoe motion sensor (IMS), we first determined predictors from specific gait phases associated with HGS, called IMS predictors. Demographic data, including age, height, weight, and BMI (IPA predictors) and a number of gait parameters (GP predictors) were also used as auxiliary predictors to improve model precision. We selected the optimal predictor combination to construct optimal estimation model M_o via our original leave-one-subject-out least absolute shrinkage and selection operator (LOSO-LASSO) process (Fig. 3). To compare the effect of our method, especially the effect of the IMS predictors, models were derived from three other patterns of predictor combinations. M_1: gait speed (GP02), M_2: M_1 plus other GPs in one stride, and M_3: M_2 plus IPAs were also constructed and evaluated for comparison. Here, M_1 had only one predictor; thus, we directly conducted linear regression. M_2 and M_3 were optimized by the same process as M_o, as shown in Fig. 3(b). For each case, in the same way as M_o, all candidate models H_1–H_{100} were also evaluated by leave-one-subject-out cross-validation (LOSOCV), and type (2, 1) of the intra-class correlation coefficient [ICC(2, 1)] was applied as the evaluation index. Each H_i had an ICC(2, 1) value, and the one with the highest AIC value was chosen as the optimal one.

The results showing the optimal predictor combination in M_2 and M_3 are shown in Figs. S1(a) and (b). The predictors selected for M_2 and M_3 are listed in Table S1.

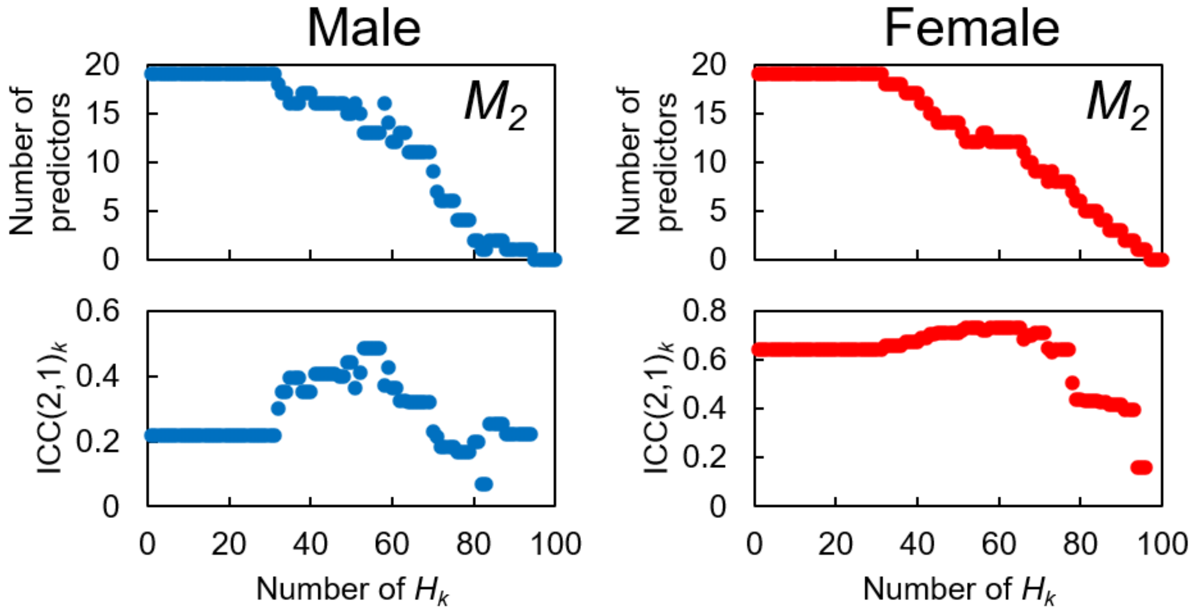

(**a**)

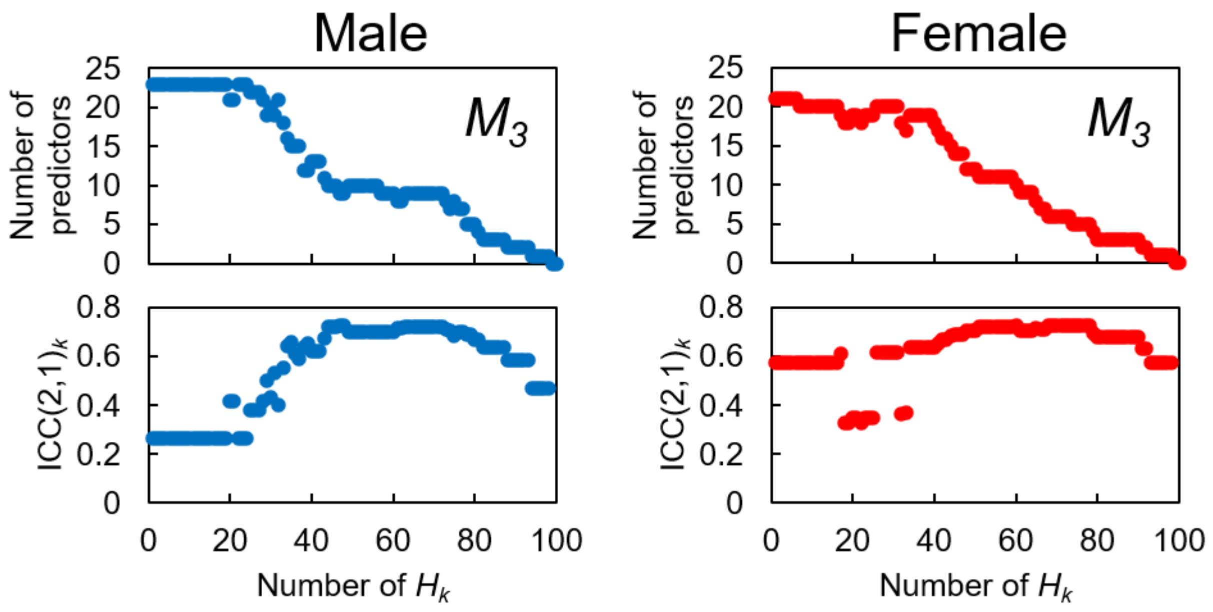

(b)

Figure S1. Results of LOSO-LASSO analysis to determine (a) M_2 and (b) M_3.

Table S1. Optimal predictor combination in M_2 and M_3.

	Male	Female
M_2	GP01–03, GP05–08, GP10, GP13, GP15–17, GP20	GP01–03, GP06–10, GP13, GP15, GP16, GP20
M_3	Age, Weight, GP05, GP07, GP08, GP10, GP15, GP16, GP20	Age, Height, GP03, GP07, GP13, GP16

[1] Please note that GP01, GP05, and GP06 were normalized by subject height. GP11–14, GP19, and GP20 were normalized by time duration of one stride. GP15, GP16, and GP18 were normalized by maximum instantaneous walking velocity during swing phase.

2. Correlation analysis between designed frailty risk score and expert-rated score in other patterns

The correlations between the expert-rated median score and the three types of performance scores calculated from the reference value and IMS-estimated value are shown in Fig. S2. The expert-rated median score was significantly correlated with the P_{HGS}, P_{GS}, and P_{fr} with large effect sizes. The rs of P_{GS} and P_{fr} were higher than the pattern using the mean expert-rated score. In this study, for the mean expert-rated score of all subjects, the six values followed a normal distribution, but for several specific subjects, there were outliers in the six values. Therefore, rs may be increased by the excluding the outliers for these subjects.

The correlations between the expert-rated mean rank and the three types of performance scores calculated from the reference and IMS-estimated values are shown in Fig. S3. The rank reflected the relative frailty status of subjects in the cohort. The performance scores also had a large size effect correlation coefficient with the expert-rated mean rank. In addition, the rs of all three types of performance scores were higher than the pattern using the mean expert-rated score. However, please note that the rank was only meaningful for the cohort in this study; it cannot be expanded to other cohorts.

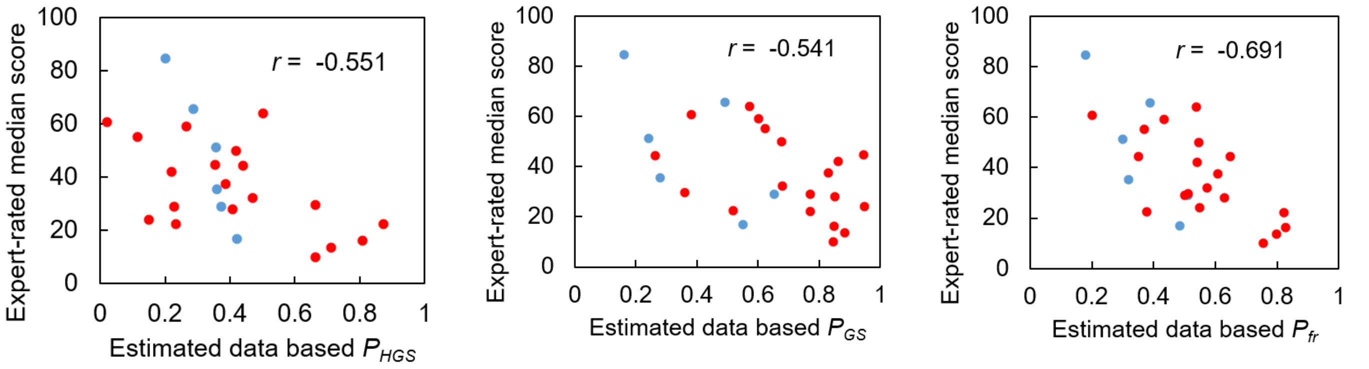

(**a**) (**b**) (**c**)

Figure S2. Correlations between expert-rated median score and three types of performance scores calculated from IMS-estimated value: (**a**) P_{HGS}, (**b**) P_{GS}, (**c**) P_{fr}.

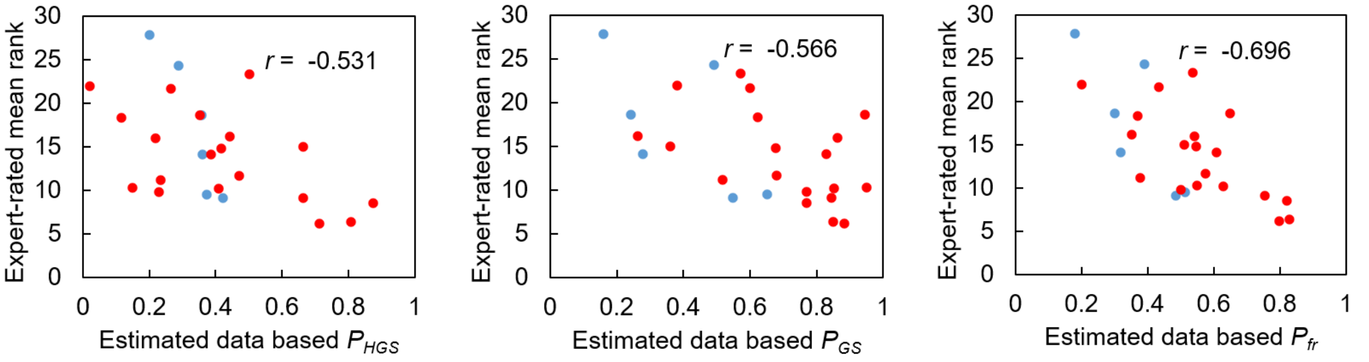

(**a**) (**b**) (**c**)

Figure S3. Correlations between expert-rated mean rank and three types of performance scores calculated from IMS-estimated value: (**a**) P_{HGS}, (**b**) P_{GS}, (**c**) P_{fr}.
